# *Trichoderma* collection from Brazilian soil reveals a new species: *T. cerradensis* Sp. nov.

**DOI:** 10.3389/fmicb.2025.1279142

**Published:** 2025-02-11

**Authors:** Gustavo Henrique Silva Peixoto, Rildo Alexandre Fernandes da Silva, Ana Beatriz Zacaroni, Thais França Silva, Priscila Chaverri, Danilo Batista Pinho, Sueli Corrêa Marques de Mello

**Affiliations:** ^1^Universidade de Brasília, Brasília, Brazil; ^2^Embrapa Recursos Genéticos e Biotecnologia, Brasília, Brazil; ^3^Department of Natural Sciences, Bowie State University, Bowie, MD, United States; ^4^Escuela de Biología and Centro de Investigaciones en Productos Naturales CIPRONA, Universidad de Costa Rica, San José, Costa Rica

**Keywords:** Hypocreales, Hypocreaceae, multigenic, new taxon, biological control

## Abstract

*Trichoderma* spp. are important biological control agents and plant growth promoters. However, only a limited number of species are used in biological control even though the genus contains more than 400 species, with most of them being mycotrophic. In this study, 97 *Trichoderma* isolates preserved at the EMBRAPA collection (an important source for biocontrol agents) and previously collected from several areas in Brazil were characterized which were identified using various molecular markers (internal transcribed spacers (*its*), translation elongation factor (*tef1α*), RNA polymerase II subunit (*rpb2*), actin (*act*), and calmodulin (*cal*). Of these, 54 isolates were found to group in the *Harzianum* species complex and 32 in Sect. *Trichoderma*. Others were distributed in the following clades: *Strictipilosa* complex (one isolate), *Longibrachiatum* (four isolates), and *Brevicompactum* (seven isolates). Most of the isolates were identified within 17 known species, whereas *Trichoderma inhamatum* and *T. dorothopsis* were synonymized under *T. lentiforme* and *T. koningiopsis*, respectively, based on multi-locus phylogenetic analysis and GCPRS criteria. However, two isolates formed a clade apart from previously identified species from Sect. *Trichoderma* and identified as a new species: *T. cerradensis* sp. nov. The multigenic characterization of isolates deposited in fungal culture collections is crucial for accurate identification and reveals a diverse range of *Trichoderma* species in Brazil.

## 1 Introduction

*Trichoderma* Persoon (1794) (=*Hypocrea*) contains mycotrophic and saprotrophic fungal species that can be found in diverse habitats such as leaf-cutting ant nests, soil, rhizosphere, decomposing plant material, fungal sporocarps, and as endophytes, and in multiple geographic regions, from the Arctic to the Tropics (Chaverri and Samuels, 2003; Hughes et al., 2007; Montoya et al., 2016). These fungi exhibit a myriad of applications, owing to the diverse array of research fields that have utilized them over the past century (Dou et al., 2020). Several species are being studied for use in environmental bioremediation processes and production of heterologous enzymes of scientific and industrial interest (Tomico-Cuenca et al., 2021; Mukherjee et al., 2013). However, this genus is especially important in agriculture as biological control agents and plant growth promoters and is known to increase drought tolerance in plants (Weindling, 1932, 1934; López-Bucio et al., 2015; Mukesh et al., 2016; Ben M’henni et al., 2022). A few species have been reported to cause human diseases, especially in immunocompromised patients, while others are etiologic agents of green mold disease in mushroom cultivation (Chen and Zhuang, 2017).

The cosmopolitan nature of *Trichoderma* is evident in its widespread distribution. Its species can be present in the most diverse habitats, almost without restriction of environmental conditions. Studies have reported their ability to survive in various geographical areas of the planet and on different continents, i.e., Africa (Allsopp et al., 1987; du Plessis et al., 2018; del Carmen et al., 2021), Oceania (Jaklitsch and Voglmayr, 2014), Europe (Jaklitsch, 2009), Asia (Qiao et al., 2018), and America (Chaverri et al., 2015; Almeida et al., 2018; Hanada et al., 2008; Bustamante et al., 2021). There are also reports of the presence of *Trichoderma* in extreme conditions, such as *T. koningii* in Antarctic soil (Hughes et al., 2007), *T. viride* in ice tunnels at the North Pole (Jacobs et al., 1964), and in highly polluted environments or in kerosene tanks (Klein and Eveleigh, 1998; Druzhinina et al., 2012).

Even though Brazil is one of the most important biodiversity hotspots, few studies have assessed and characterized its *Trichoderma* diversity. One study identified many *Trichoderma* species from soil samples of garlic and onion (Inglis et al., 2020). Recently, four new species were described from Brazilian Amazon (Brito et al., 2023), while several isolates were characterized with sequences of the translation elongation factor 1-*α* gene region (Oliveira et al., 2023). Therefore, it is difficult to estimate the number of species present in Brazil. In a study carried out in China by Hu et al. (2020), covering 1,236 samples collected from 40 locations with diverse climatic conditions and ecosystems, 919 isolates belonging to 39 species in 9 complexes were identified, in addition to revealing another 317 isolates as potentially new species. Another study conducted in Europe with more than 650 isolates revealed greater genetic variation than previously observed. These authors found 96 species, among which 17 were considered new species (Jaklitsch and Voglmayr, 2015). Studies with this impact are important to know the variability and distribution of fungi of this genus, in addition to providing information on the ideal environmental conditions for survival of each species. Furthermore, the correct identification of the species may relate to their biological applications (Hoyos-Carvajal and Bisset, 2011).

Through sequencing of actin (*act*), calmodulin (*cal*), internal transcribed spacers (*its*), RNA polymerase II subunit 2 (*rpb2*), and translation elongation factor 1-*α* (*tef1α*), commercial isolates registered as *T. harzianum* in North America and Europe were reanalyzed by Chaverri et al. (2015). These authors concluded that such products were, in fact, *T. afroharzianum*, *T. guizhouense*, and *T. simmonsii*. No isolates of *Trichoderma harzianum sensu stricto* were identified among the analyzed samples. In Brazil, there are 21 *Trichoderma*-based biofungicides available, and 14 of these were registered as *T. harzianum*, 5 as *T. asperellum*, 1 as *T. koningiopsis*, and 1 as *T. stromaticum* (Bettiol et al., 2012, 2019). According to the product labels, *T. harzianum* is present as an active component in 38.8% of the products, 50% when mixed with other *Trichoderma* species, and up to 60% if considered in a mixture with other microorganisms (Bettiol et al., 2019). This confusion in the identification of *Trichoderma* species may be underestimated, considering the products currently registered as *T. harzianum* and the mistaken identification in other countries (Chaverri et al., 2015; Bettiol et al., 2019). Probably, the identification of such isolates occurred at times before the emergence of modern sequencing techniques and multi-loci analysis and was based primarily on morphological characteristics. Therefore, a review of the identification and the reclassification of these isolates is urgently needed. Especially in the case of species that will compose new commercial products, at least the *tef1α* and *rpb2* (Atanasova et al., 2013) should be used to confidently identify the active ingredients. In view of the above, the objective of this study was to correctly identify and characterize the *Trichoderma* isolates kept in the Empresa Brasileira de Pesquisa Agropecuaria (EMBRAPA) collection, an important source of biocontrol agents, which were previously isolated from different locations in Brazil.

## 2 Materials and methods

### 2.1 Source of isolates

Ninety-seven *Trichoderma* isolates from the EMBRAPA Biological Control Agents Collection were used (Table 1). The isolates were collected from different geographical areas of Brazil. The cultures were kept in liquid nitrogen. For this study, they were reactivated in potato dextrose agar (PDA) and then stored for a short period at 10°C in test tubes containing 20 mL of PDA.

**Table 1:** *Trichoderma* species from biological control collection on EMBRAPA genetic resources and biotechnology characterized in this study.

Species	CEN	Geographical area	Rizosphere/host	Complex
*T. brevicompactum*	CEN510	Pernambuco State	*Psidium guajava*	*Brevicompactum*
*T. brevicompactum*	CEN1071	Federal District	*Solanum lycopersicum*	*Brevicompactum*
*T. brevicompactum*	CEN1074	Federal District	*Solanum lycopersicum*	*Brevicompactum*
*T. brevicompactum*	CEN1245	Federal District	*Solanum lycopersicum*	*Brevicompactum*
*T. brevicompactum*	CEN1274	Federal District	*Brassica oleracea*	*Brevicompactum*
*T. brevicompactum*	CEN1300	Federal District	*Abelmoschus esculentus*	*Brevicompactum*
*T. brevicompactum*	CEN1544	Mato Grosso State	Pantanal Biome	*Brevicompactum*
*T. afroharzianum*	CEN254	Federal District	*Gossypium* sp.	*Harzianum*
*T. afroharzianum*	CEN281	Federal District	*Gossypium* sp.	*Harzianum*
*T. afroharzianum*	CEN287	Federal District	*Gossypium* sp.	*Harzianum*
*T. afroharzianum*	CEN289	Federal District	*Gossypium* sp.	*Harzianum*
*T. peberdyi*	CEN256	Federal District	*Gossypium* sp.	*Harzianum*
*T. rifaii*	CEN288	Federal District	*Gossypium* sp.	*Harzianum*
*T. rifaii*	CEN290	Federal District	*Gossypium* sp.	*Harzianum*
*T. rifaii*	CEN298	Federal District	*Gossypium* sp.	*Harzianum*
*T. rifaii*	CEN316	Federal District	*Gossypium* sp.	*Harzianum*
*T. afarasin*	CEN141	Goiás State	*Glycine max*	*Harzianum*
*T. afroharzianum*	CEN155	Goiás State	*Zea mays*	*Harzianum*
*T. afroharzianum*	CEN158	Goiás State	*Oryza sativa*	*Harzianum*
*T. afroharzianum*	CEN197	Goiás State	*Sorghum bicolor*	*Harzianum*
*T. afroharzianum*	CEN230	Federal District	*Gossypium* sp.	*Harzianum*
*T. afroharzianum*	CEN234	Federal District	*Gossypium* sp.	*Harzianum*
*T. afroharzianum*	CEN235	Federal District	*Gossypium* sp.	*Harzianum*
*T. afroharzianum*	CEN1059	Rio Grande do Sul State	Pampa Biome	*Harzianum*
*T. afroharzianum*	CEN1249	Federal District	*Solanum lycopersicum*	*Harzianum*
*T. afroharzianum*	CEN1328	Federal District	*Capsicum annuum*	*Harzianum*
*T. afroharzianum*	CEN1417	São Paulo State	*Allium cepa*	*Harzianum*
*T. afroharzianum*	CEN1546	Mato Grosso State	Pantanal Biome	*Harzianum*
*T. austroindianum*	CEN1555	Mato Grosso State	Pantanal Biome	*Harzianum*
*T. austroindianum*	CEN1561	Mato Grosso State	Pantanal Biome	*Harzianum*
*T. azevedoi*	CEN168	Goiás State	*Sorghum bicolor*	*Harzianum*
*T. azevedoi*	CEN1069	Federal District	*Solanum lycopersicum*	*Harzianum*
*T. azevedoi*	CEN1242	Federal District	*Zea mays*	*Harzianum*
*T. azevedoi*	CEN1250	Federal District	*Solanum lycopersicum*	*Harzianum*
*T. azevedoi*	CEN1282	Federal District	*Spinacia oleracea*	*Harzianum*
*T. azevedoi*	CEN1293	Federal District	*Tibouchina* sp.	*Harzianum*
*T. azevedoi*	CEN1304	Federal District	*Cucurbita pepo*	*Harzianum*
*T. azevedoi*	CEN1325	Federal District	*Zea mays*	*Harzianum*
*T. hortense*	CEN1243	Federal District	*Solanum melongena*	*Harzianum*
*T. hortense*	CEN1515	Amazonas State	Amazonic Rainforest	*Harzianum*
*T. lentiforme*	CEN223	Federal District	*Gossypium* sp.	*Harzianum*
*T. lentiforme*	CEN1153	Mato Grosso State	*Tectona grandis*	*Harzianum*
*T. lentiforme*	CEN1294	Federal District	*Zanthoxylum rhoifolium*	*Harzianum*
*T. lentiforme*	CEN1336	Federal District	*Miconia elegans*	*Harzianum*
*T. lentiforme*	CEN1416	São Paulo State	*Allium cepa*	*Harzianum*
*T. peberdyi*	CEN198	Federal District	*Gossypium* sp.	*Harzianum*
*T. peberdyi*	CEN211	Federal District	Cerrado Biome	*Harzianum*
*T. peberdyi*	CEN225	Federal District	*Gossypium* sp.	*Harzianum*
*T. peberdyi*	CEN226	Federal District	*Gossypium* sp.	*Harzianum*
*T. peberdyi*	CEN228	Federal District	*Gossypium* sp.	*Harzianum*
*T. peberdyi*	CEN232	Federal District	*Gossypium* sp.	*Harzianum*
*T. rifaii*	CEN202	Federal District	*Gossypium* sp.	*Harzianum*
*T. rifaii*	CEN238	Federal District	*Gossypium* sp.	*Harzianum*
*T. rifaii*	CEN239	Federal District	*Gossypium* sp.	*Harzianum*
*T. rifaii*	CEN240	Federal District	*Gossypium* sp.	*Harzianum*
*T. rifaii*	CEN242	Goiás State	*Oryza sativa*	*Harzianum*
*T. rifaii*	CEN1263	Federal District	*Solanum melongena*	*Harzianum*
*T. rifaii*	CEN1267	Federal District	*Zea mays*	*Harzianum*
*Trichoderma* sp.	CEN1283	Federal District	*Tithonia diversifolia*	*Harzianum*
*Trichoderma* sp.	CEN1351	Federal District	*Cyathea* sp.	*Harzianum*
*T. ghanense*	CEN555	Bahia State	Cerrado Biome	*Longibrachiatum*
*T. ghanense*	CEN1550	Mato Grosso State	Pantanal Biome	*Longibrachiatum*
*T. longibrachiatum*	CEN1281	Federal District	*Zea mays*	*Longibrachiatum*
*T. longibrachiatum*	CEN1562	Mato Grosso State	Pantanal Biome	*Longibrachiatum*
*T. asperelloides*	CEN162	Goiás State	*Oryza sativa*	Sect. *Trichoderma*
*T. asperelloides*	CEN277	São Paulo State	Atlantic Forest Biome	Sect. *Trichoderma*
*T. asperelloides*	CEN1276	Federal District	*Brassica oleracea*	Sect. *Trichoderma*
*T. asperelloides*	CEN1277	Federal District	*Petroselinum crispum*	Sect. *Trichoderma*
*T. asperelloides*	CEN1338	Federal District	*Miconia elegans*	Sect. *Trichoderma*
*T. asperelloides*	CEN1343	Federal District	*Zea mays*	Sect. *Trichoderma*
*T. asperelloides*	CEN1354	Federal District	*Cestrum* sp.	Sect. *Trichoderma*
*T. asperelloides*	CEN1514	Amazonas State	Amazonic Rainforest	Sect. *Trichoderma*
*T. asperelloides*	CEN1532	Federal District	*Glycine max*	Sect. *Trichoderma*
*T. asperelloides*	CEN1533	Federal District	*Glycine max*	Sect. *Trichoderma*
*T. asperelloides*	CEN1542	Mato Grosso State	Pantanal Biome	Sect. *Trichoderma*
*T. asperelloides*	CEN1559	Mato Grosso State	Pantanal Biome	Sect. *Trichoderma*
*T. asperellum*	CEN201	Tocantins State	*Vochysia* sp.	Sect. *Trichoderma*
*T. asperellum*	CEN698	Federal District	*Fragaria × ananassa*	Sect. *Trichoderma*
*T. asperellum*	CEN768	Federal District	*Fragaria × ananassa*	Sect. *Trichoderma*
*T. asperellum*	CEN1075	Federal District	*Solanum lycopersicum*	Sect. *Trichoderma*
*T. subviride*	CEN144	Goiás State	Cerrado Biome	Sect. *Trichoderma*
*T. atroviride*	CEN875	Rio Grande do Sul State	Pampa Biome	Sect. *Trichoderma*
*T. erinaceum*	CEN1558	Mato Grosso State	Pantanal Biome	Sect. *Trichoderma*
*T. hamatum*	CEN1334	Federal District	*Manihot esculenta*	Sect. *Trichoderma*
*T. hamatum*	CEN1350	Federal District	*Manihot esculenta*	Sect. *Trichoderma*
*T. koningiopsis*	CEN203	Federal District	*Gossypium* sp.	Sect. *Trichoderma*
*T. koningiopsis*	CEN209	Federal District	*Copaifera langsdorffii*	Sect. *Trichoderma*
*T. koningiopsis*	CEN865	Rio Grande do Sul State	Pampa Biome	Sect. *Trichoderma*
*T. koningiopsis*	CEN980	Rio Grande do Sul State	Pampa Biome	Sect. *Trichoderma*
*T. koningiopsis*	CEN1257	Federal District	*Manihot esculenta*	Sect. *Trichoderma*
*T. koningiopsis*	CEN1265	Federal District	*Cyathea* sp.	Sect. *Trichoderma*
*T. koningiopsis*	CEN1301	Federal District	*Solanum lycopersicum*	Sect. *Trichoderma*
*T. koningiopsis*	CEN1333	Federal District	*Manihot esculenta*	Sect. *Trichoderma*
*T. koningiopsis*	CEN1513	Amazonas State	*Theobroma cacao*	Sect. *Trichoderma*
*T. cerradensis* sp. nov.	CEN221	Federal District	*Gossypium* sp.	Sect. *Trichoderma*
*T. cerradensis* sp. nov.	CEN1348	Federal District	*Cucurbita pepo*	Sect. *Trichoderma*
*T. spirale*	CEN1247	Federal District	*Cucurbita pepo*	*Strictipilosa*

### 2.2 DNA extraction, PCR, and sequencing

The isolates were grown in 50 mL Falcon tubes containing potato dextrose broth (PDB) medium (at 25°C for 5 days on a shaker Adicione one). Then, the mycelium was collected with a sterile toothpick and deposited in 1.5 mL microtubes containing 20 μL of Tris-EDTA (TE) buffer. DNA extraction was done using a Wizard Genomic DNA Purification Kit (Promega®) according to the protocol adapted by Pinho et al. (2012). The presence and the quality of genomic DNA were assessed in 1% agarose gel electrophoresis, stained with GelRed (Biotium®), and visualized under UV light. The genomic DNA was stored at −20°C for later use.

Part of the gene encoding translation elongation factor 1-alpha (*TEF1α*; ca. 700 bp) was amplified and sequenced for a preliminary identification as it is considered a useful secondary barcode (Druzhinina et al., 2012; Chaverri et al., 2015). After this preliminary identification, internal transcribed spacers of the nuclear ribosomal DNA (*its*, ca. 1,500 bp), RNA polymerase II (*rpb2*; ca. 900 bp), calmodulin (*cal*; 700 bp), and actin (*act*; 700 bp) were sequenced for selected isolates. The primers used are listed in Table 2. Polymerase chain reactions (PCRs) were performed in a final volume of 12.5 μL: 6.25 μL of MyTaq MasterMix 2x (Bioline, EUA), 0.3 μL (10 pmol/μL) of each primer, 4.25 μL of nuclease-free water, and 1 μL of template DNA (25 ng/μL). The cycle conditions for all markers were as follows: initial denaturation at 96°C for 5 min followed by 30 cycles at 90°C for 30 s, annealing temperature (according to Table 2) for 45 s, and 72°C for 45 s, and a final extension at 72°C for 5 min. The PCR products were purified and bidirectionally Sanger-sequenced.

**Table 2:** Primers selected for *Trichoderma* species identification by phylogenetic analyses.

Genic region	Initiator	Sequence	Sense	References	Amplicon length	Annealing temperature
18S-5.8 S-28S rDNA (ITS)	LR5	TCCTGAGGGAAACTTCG	Sense	Vilgalys and Hester (1990)	1,500 bp	53°C
	V9G	TTACGTCCCTGCCCTTTGTA	Antisense	De Hoog and Van Den Ended (1998)		
Translation elongation factor (TEF1α)	EF1F	TGCGGTGGTATCGACAAGCGT	Sense	Jacobs et al. (2004)	700 bp	56°C
	EF2R	AGCATGTTGTCGCCGTTGAAG	Antisense	Jacobs et al. (2004)		
Calmodulin (*cal*)	CAL-228F	GAGTTCAAGGAGGCCTTCTCCC	Sense	Carbone and Kohn (1999)	700 bp	55°C
	Cal2RD	TGRTCNGCCTCDCGGATCATCTC	Antisense	Groenewald et al. (2013)		
Actin (*act*)	TRI-ACT1	TGGCACCACACCTTCTACAATGA	Sense	Samuels et al. (2006)	700 bp	56°C
	TRI-ACT2	TCTCCTTCTGCATACGGTCGGA	Antisense	Samuels et al. (2006)		
RNA polymerase II (RPB2)	5F2	GGGGWGAYCAGAAGAAGGC	Sense	Sung et al. (2007)	1,100 bp	57°C
	7cR	CCCATRGCTTGYTTRCCCAT	Antisense	Liu et al. (1999)		

### 2.3 Phylogenetic analyses

The quality of the sequences and contig assembly were checked, with subsequent ambiguity analysis, and, when necessary, adjusted by comparing the sense and antisense strands through DNA Dragon software.^1^ The TEF1α sequences were submitted to the BLASTn algorithm (Altschul et al., 1990) within the NCBI platform and compared with sequences downloaded from the GenBank nucleotide database^2^ found in publications to determine the origin of the species complex. Phylogenetic analyses were first performed for each gene separately. Then, multi-loci analyses were performed for each *Trichoderma* clade separately (e.g., *Harzianum*, *Longibrachiatum*, *Brevicompactum*, sect. *Trichoderma*, and *Strictipilosa*). Sequences were aligned in MAFFT v.7 (Katoh and Standley, 2013) and manually refined in MEGA v.7 software (Kumar et al., 2016). For Bayesian inference (BI), the best nucleotide substitution models for each partition were determined with MrModeltest 2.3 (Nylander, 2004). The model selected for maximum likelihood (ML) analysis was GTR + G (Stamatakis, 2014). The models were added to a command block in each corresponding matrix, which was later concatenated into a single supermatrix. To construct phylogenetic trees, MrBayes 3.1.2 was used for BI (Ronquist and Huelsenbeck, 2003) and RAxML-HPC2 8.2.12 for ML (Stamatakis, 2014) within the CIPRES Portal (Miller et al., 2010). For BI, 10 million generations were run, with sampling every 1,000 and subsequent removal of the 25% first trees in the analysis (burn-in), followed by the assembly of consensus tree using the 7,500 remaining trees and calculation of posterior probability (PP). The convergence of the log likelihoods was confirmed using TRACER v1.7.1 (Rambaut and Drummond, 2018). For ML, 1,000 bootstrap (BS) replicates were used. The trees were visualized using FigTree v.1.4 (Rambaut, 2018) and edited in graphics programs. For species attribution, the criteria of Genealogical Concordance Phylogenetic Species Recognition (GCPSR) were employed. The criteria of genealogical concordance, which is satisfied when the clade is present in the majority of individual trees, and genealogical non-discordance, which is achieved when the clade is strongly supported (≥70% for ML and ≥ 0.95 for BI), were analyzed. The new species and their synonymization were recognized when the GCPSR criteria were met.

### 2.4 Morphological characterization

The morphological characteristics of the colonies were determined only for the isolate CEN221, the holotype of *T. cerradensis* sp. nov. The growth trials were done in 90 mm diameter Petri dishes, containing 20 mL of PDA, cornmeal dextrose (CMD) agar, and synthetic nutrient agar (SNA) medium without a filter paper (Nirenberg, 1976), according to Chaverri et al. (2015). The cultures were incubated at 25°C under alternating 12-h/12-h light/darkness. Growth rate measurements (radius in mm) were recorded daily for 5 days. Culture characteristics were observed after 7 days. The micromorphological characteristics were performed based on a microculture technique, from 3-day-old colonies grown on SNA at 25°C, and conidia and conidiophores were analyzed by mounting semi-permanent slides in lactoglycerol. Thirty-five measurements for each of these morphological parameters were done at a magnification of ×1,000, using a Leica DM2500 light microscope equipped with a Leica DFC 490 digital camera, coupled to a computer containing Leica Qwin-Plus software. The average, standard deviation, and maximum and minimum values were calculated for the measurements. The dried culture of the holotype was stored at the Brasilia University (UnB) Herbarium, while ex-type cultures preserved in liquid nitrogen were kept in the Biocontrol Agents Collection at EMBRAPA Genetic Resources and Biotechnology (CENARGEN), Brasília, Federal District.

## 3 Results

The studied isolates were distributed in the following clades: *Harzianum* complex (54 isolates), Section *Trichoderma* (32), *Brevicompactum* complex (7), *Longibrachiatum* complex (4), and *Strictipilosa* complex (1). The sequences were deposited in GenBank with the following codes: *its* (OM515005–OM515101); *tef* (ON101407–ON101503); *rpb2* (PP805906–PP854205, PQ149256–PQ149277, and PQ720596– PQ720597); *cal* (ON241149–ON241245); and *act* (ON311008–ON311104).

After analyzing all the single-gene phylogenetic trees for each complex (Supplementary material), it was observed that the *tef1α* and *rpb2* regions exhibited greater species segregation. While *cal* and *act* regions could delineate certain species, they were insufficient for comprehensive species differentiation. In addition, these two regions had the fewest sequences available for comparison in GenBank. Finally, the *its* region failed to adequately segregate species across all complexes. When focusing solely on the isolates analyzed in this study, *act*, *cal*, *rpb2*, and *tef1α* regions effectively defined the clades, whereas *its* did not perform as well.

**Figure 1:**
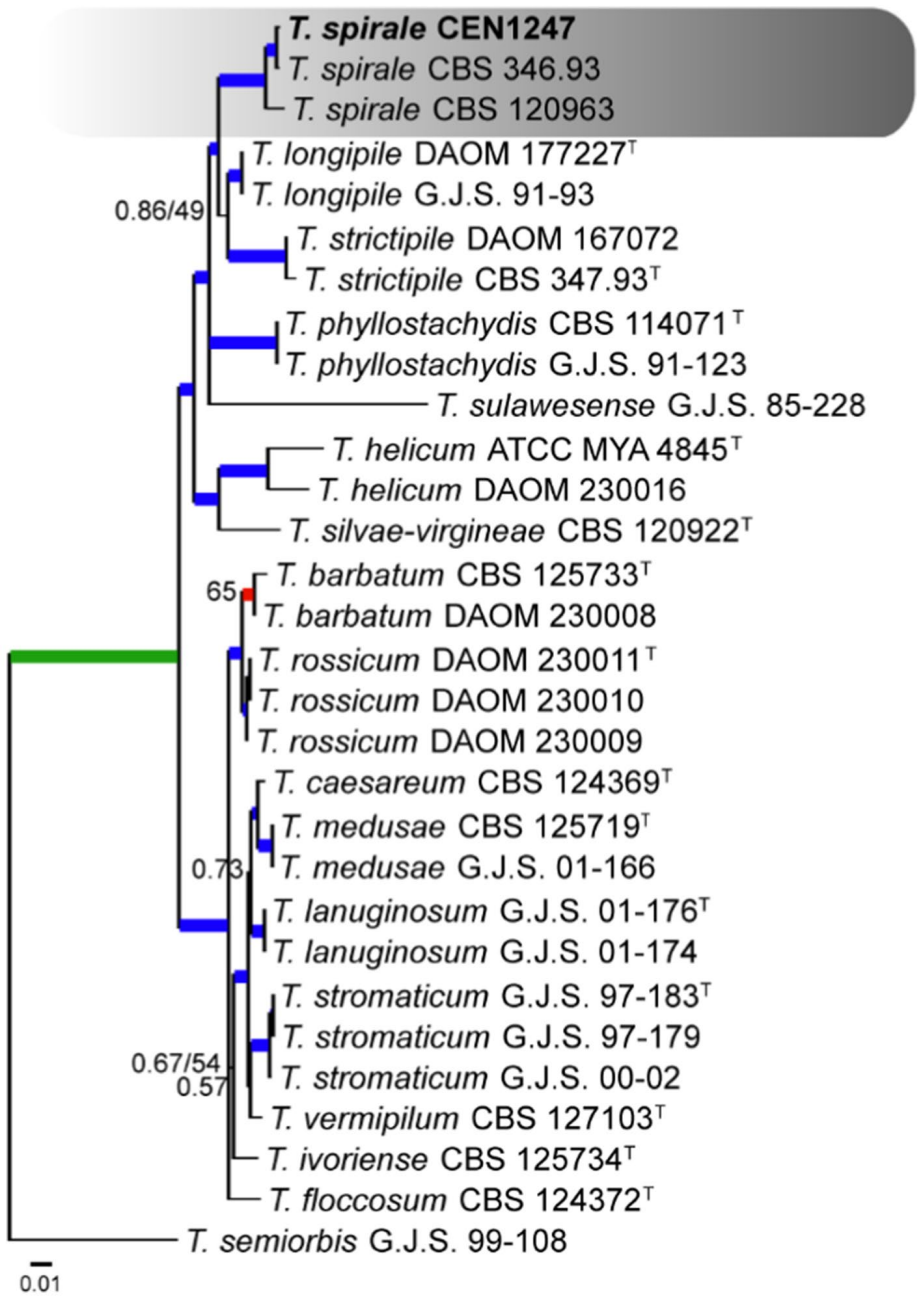
Bayesian phylogenetic tree based on concatenated sequences (*TEF1α*, *ITS*, *RPB2*, *CAL*, and *ACT*) of *Strictipilosa* and *Stromaticum* complexes. Bayesian posterior probability and maximum likelihood bootstrap support values are indicated at the nodes, and the scale bar represents the number of expected changes per site. Thickened blue lines indicate PP ≥ 0.99 and BS ≥ 95, red color indicates PP ≥ 0.99, and green color indicates BS ≥ 95. The specimen *T. semiorbis* G.J.S. 99–108 was used as the out-group. The strains reported here are highlighted in bold (T = Type specimen).

The multi-loci analysis was done for all species in each species complex. One of these, which was compared to 27 sequences available on GenBank, including accessions from *Stromaticum* clade, was shown to be part of the *Strictipilosa* complex, considering the proximity between these two species complexes. The out-group used for rooting was *T. semiorbis*. The concatenated gene matrix comprised 2,477 total characters, distributed among the *rpb2* (811 bp), *its* (602 bp), *tef1α* (548 bp), *cal* (471 bp), and *act* (716 bp) regions, including gaps. Of the total character numbers in the concatenated matrix, 2,115 sites were conserved, 968 were variable, and 477 were phylogenetically informative. The evolutionary models selected for Bayesian inference were GTR + I + G, HKY + G, GTR + I + G, K80 + G, and GTR + I for *its*, *tef1α*, *rpb2*, *cal*, and *act*, respectively. In the multi-loci tree of this complex, one isolate (CEN1247) had grouped with *T. spirale* specimens with a high PP and BS support (Figure 1).

**Figure 2:**
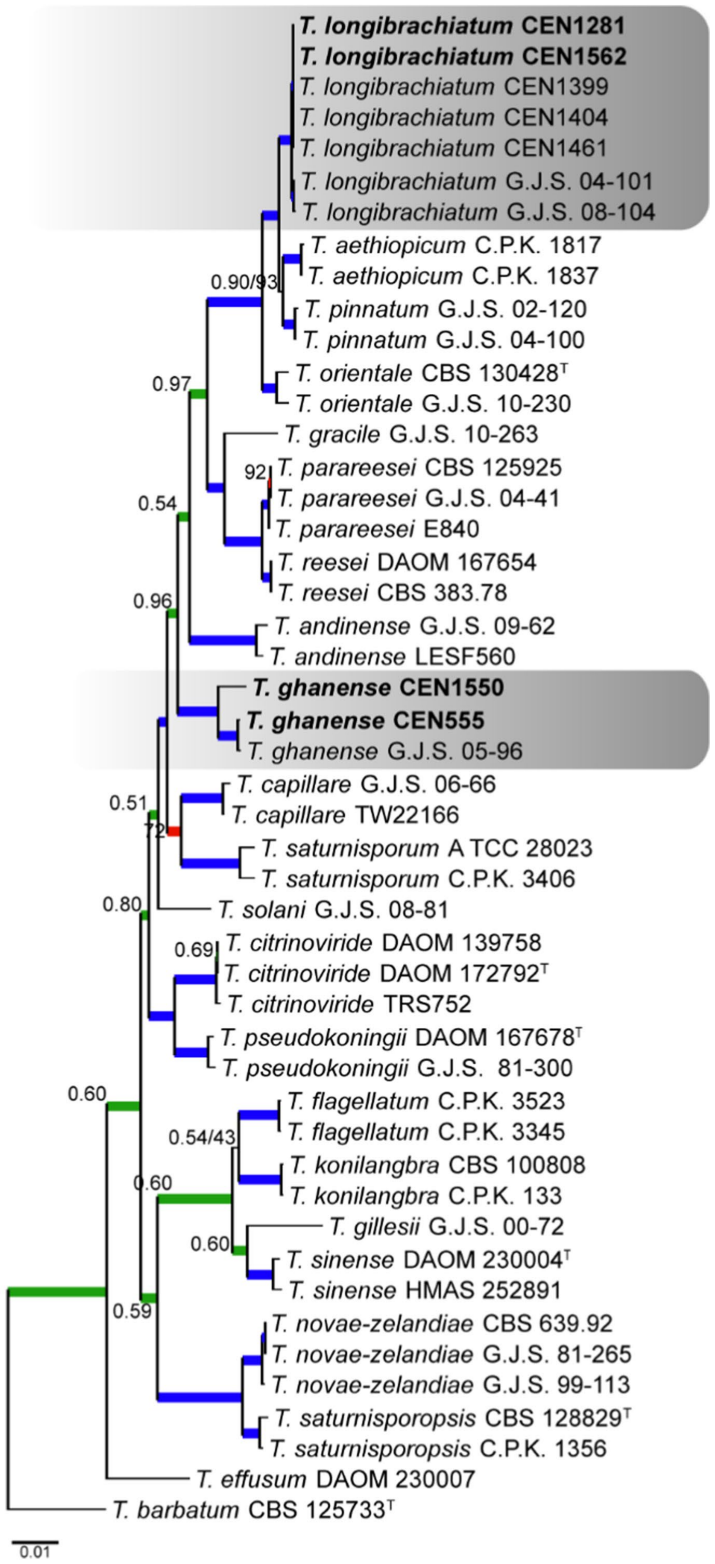
Bayesian phylogenetic tree based on concatenated sequences (*TEF1α*, *ITS*, *RPB2*, *CAL*, and *ACT*) of the *Longibrachiatum* complex. Bayesian posterior probability and maximum likelihood bootstrap support values are indicated at the nodes, and the scale bar represents the number of expected changes per site. Thickened blue lines indicate PP ≥ 0.99 and BS ≥ 95, red color indicates PP ≥ 0.99, and green color indicates BS ≥ 95. The specimen *T. barbatum* C.B.S. 125733 was used as the out-group. The strains reported here are highlighted in bold (T = Type specimen).

For the *Longibrachiatum* complex, four *Trichoderma* isolates were identified. The tree was rooted with *T. barbatum*. The concatenated matrix had 2,988 sites, distributed among *act* (718 bp), *rpb2* (703 bp), *tef1α* (590 bp), *its* (572 bp), and *cal* (400 bp), including gaps. Of these sites, 2,104 were conserved, 840 were variable, and 647 were phylogenetically informative. The selected nucleotide substitution models for *its*, *tef1α*, *rpb2*, *cal*, and *act* were GTR + I, GTR + I + G, GTR + I + G, and GTR + I + G, respectively, and GTR + I under Bayesian inference and GTR + G under maximum likelihood. It was possible to identify two isolates positioned in a clade with *T. longibrachiatum* and two others in *T. ghanense*, both with PP and BS more than 0.99 and 95%, respectively (Figure 2).

**Figure 3:**
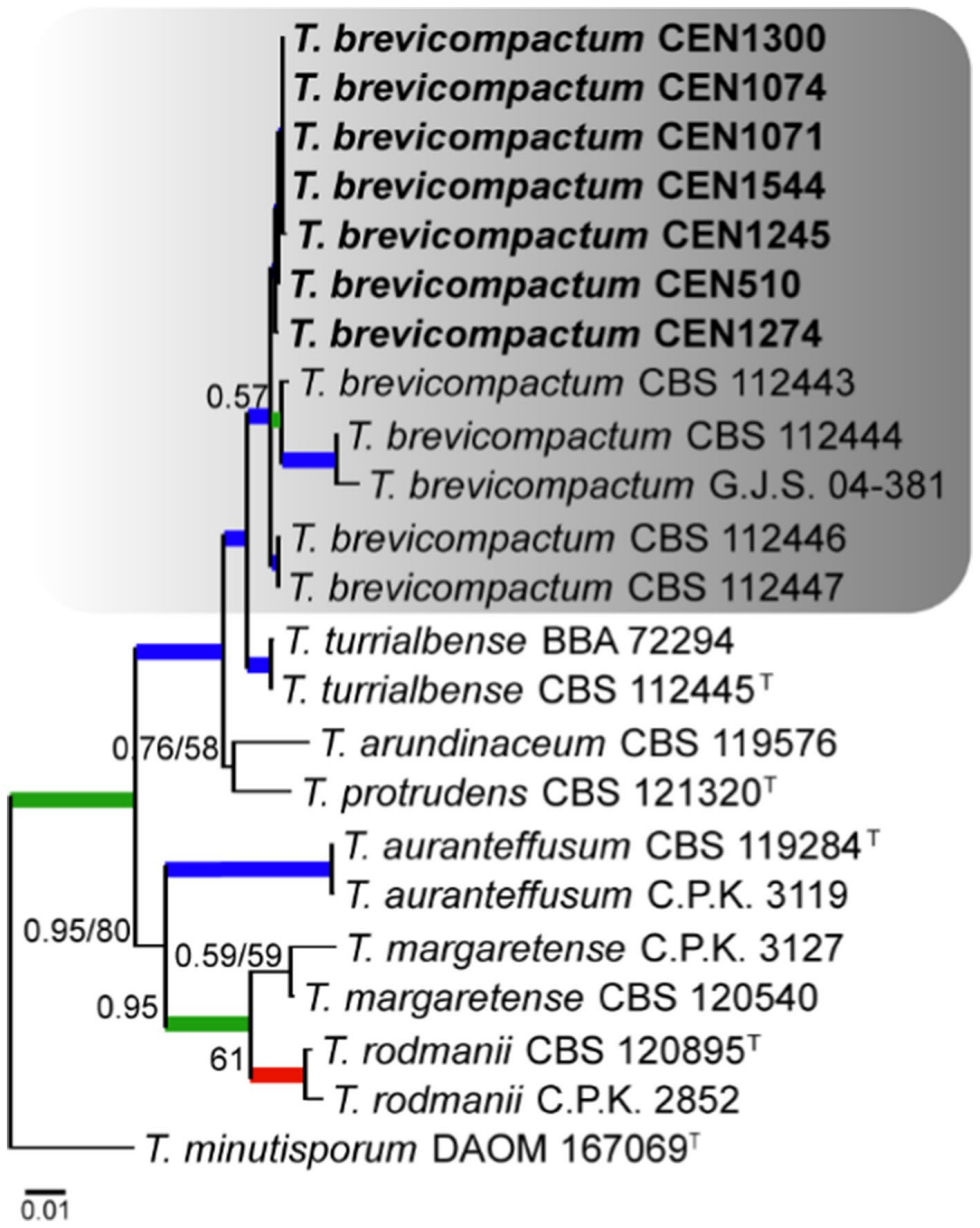
Bayesian phylogenetic tree based on concatenated sequences (*TEF1α*, *ITS*, *RPB2*, *CAL*, and *ACT*) of the *Brevicompactum* complex. Bayesian posterior probability and maximum likelihood bootstrap support values are indicated at the nodes, and the scale bar represents the number of expected changes per site. Thickened blue lines indicate PP ≥ 0.99 and BS ≥ 95, red color indicates PP ≥ 0.99, and green color indicates BS ≥ 95. The specimen *T. minutisporu*m DAOM 107069 was used as the out-group. The strains reported here are highlighted in bold (T = Type specimen).

Seven isolates were placed in the *Brevicompactum* complex, for which *T. minutisporum* was used as the out-group. The total number of characters in the concatenated matrix was 2,970, including gaps, with 751 bp for *rpb2*, 652 for *act*, 590 bp for *tef1α*, 565 bp for *its*, and 409 bp for *cal*. Of the total number of characters in the matrix, 2,374 were conserved sites, 542 were variable, and 299 were phylogenetically informative. The evolutionary models selected for Bayesian inference were HKY + I, GTR + G, SYM + I, K80, and HKY, for *its*, *tef1α*, *rpb2*, *cal*, and *act*, respectively, while GTR + G was selected for maximum likelihood. As shown in Figure 3, it was found that all seven isolates clustered close to *T. brevicompactum* with high PP and BS values.

**Figure 4:**
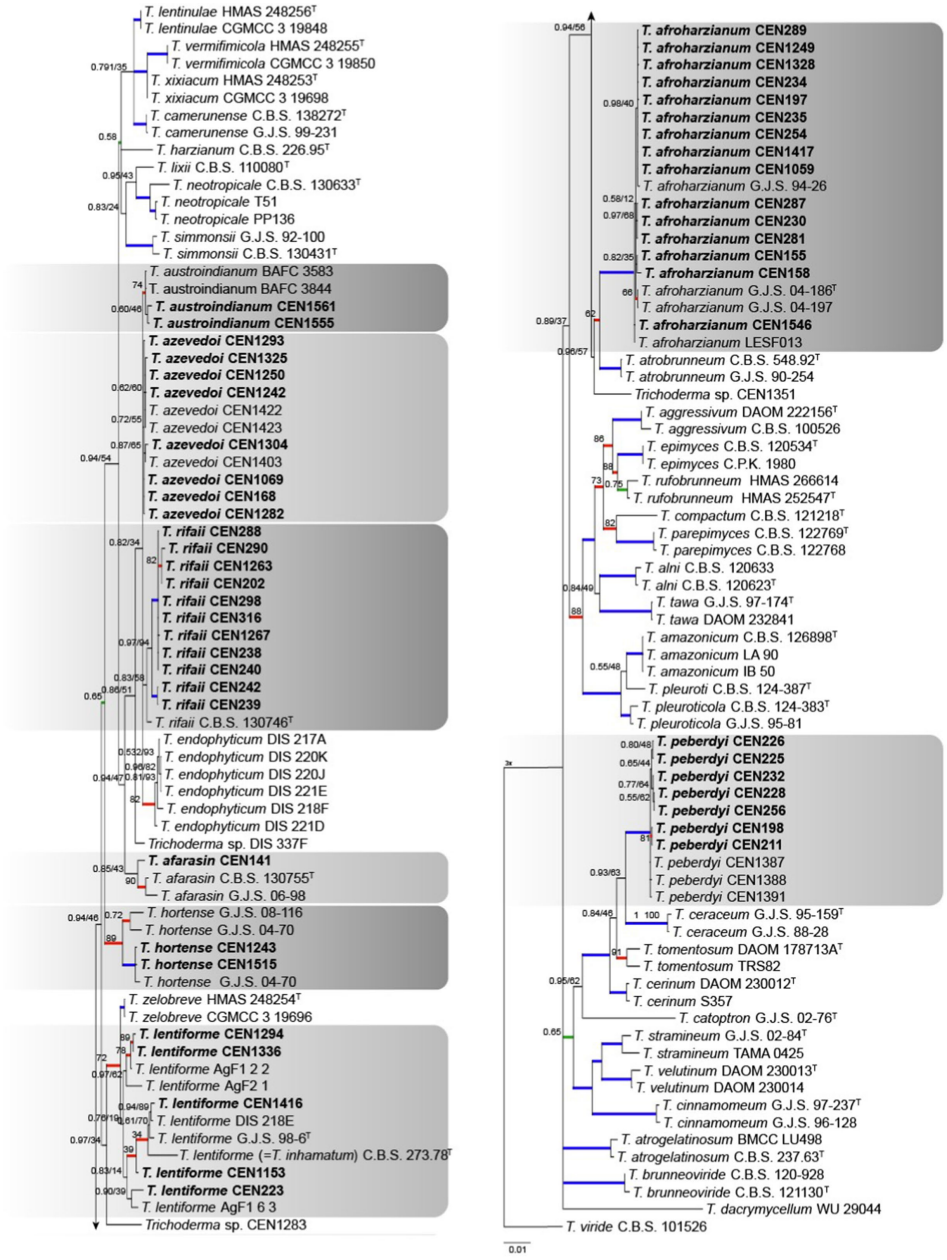
Bayesian phylogenetic tree based on concatenated sequences (*TEF1α*, *ITS*, *RPB2*, *CAL*, and *ACT*) of the *Harzianum* complex. Bayesian posterior probability and maximum likelihood bootstrap support values are indicated at the nodes, and the scale bar represents the number of expected changes per site. Thickened blue lines indicate PP ≥ 0.99 and BS ≥ 95, red color indicates PP ≥ 0.99, and green color indicates BS ≥ 95. The specimen *T. viride* CBS 101526 was used as the out-group. The strains reported here are highlighted in bold (T = Type specimen).

According to the initial screening done with the *TEF1α* region, 54 isolates grouped within the *Harzianum* complex using *T. viride* as the out-group. The concatenated matrix had 2,937 characters, with 760 for *rpb2*, 621 for *act*, 577 for *tef1α*, 528 for *its*, and 451 for *cal*. Of the total number of characters 1,940 were conserved sites, 894 were variable, and 580 were phylogenetically informative. The evolutionary models chosen for Bayesian inference were GTR + I + G, HKY + G, SYM + I + G, K80 + I + G, and GTR + I + G for *its*, *tef1α*, *rpb2*, *cal*, and *act*, respectively, and *GTR + G* for maximum likelihood. Interestingly, *T. inhamatum* CBS 273.78 was reclassified as *T. lentiforme* in our analysis. The isolates were identified to be clustering with *T. afroharzianum* (15 isolates), *T. rifaii* (11 isolates), *T. azevedoi* (8 isolates), *T. peberdyi* (7 isolates), *T. lentiforme* (5 isolates), *T. austroindianum* (2 isolates), *T. hortense* (2 isolates), and *T. afarasin* (one isolate) (Figure 4). Here, many of clades showed low PP values over 0.75.

**Figure 5:**
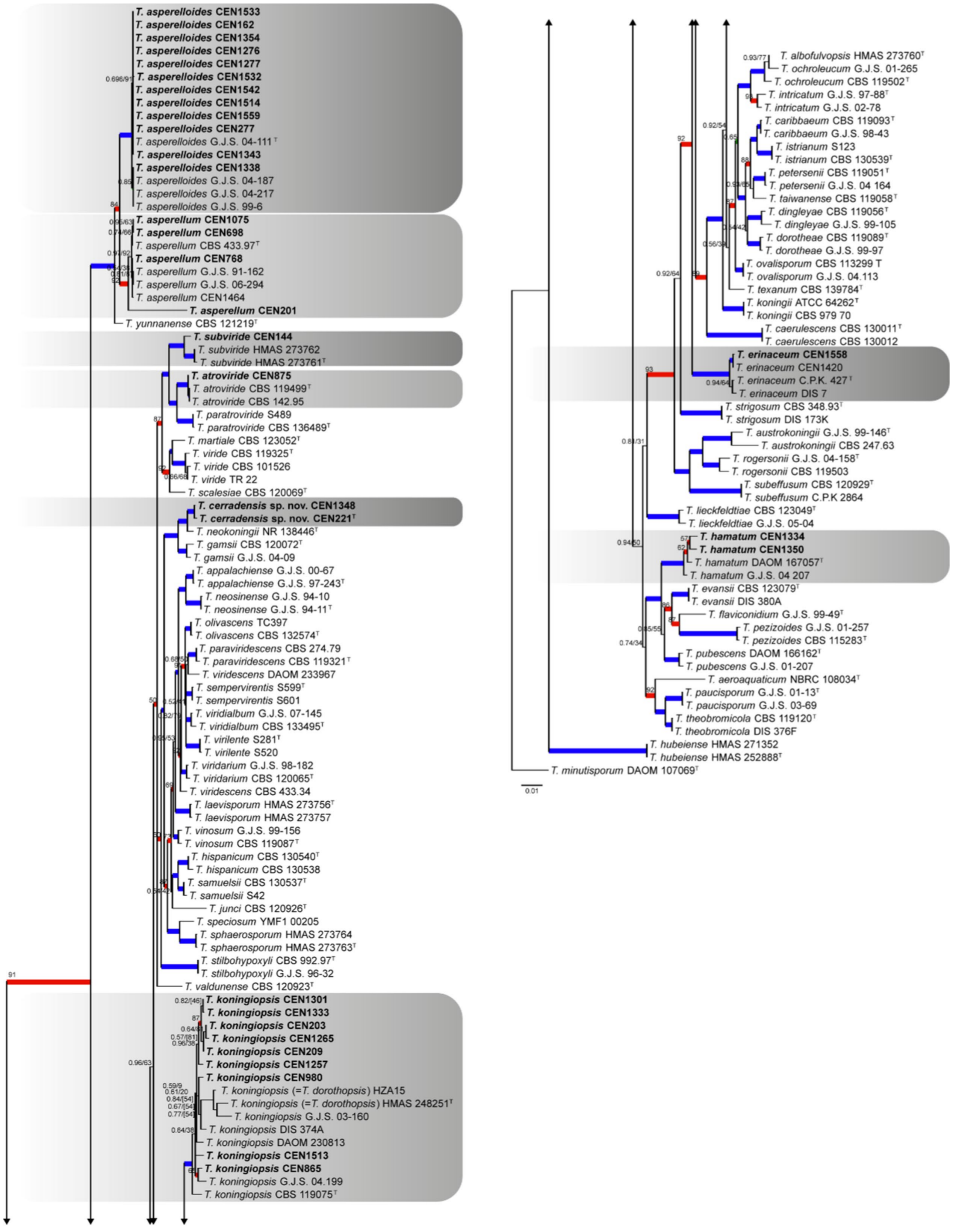
Bayesian phylogenetic tree based on concatenated sequences (*TEF1α*, *ITS*, *RPB2*, *CAL*, and *ACT*) of the Sect. *Trichoderma* complex. Bayesian posterior probability and maximum likelihood bootstrap support values are indicated at the nodes, and the scale bar represents the number of expected changes per site. Thickened blue lines indicate PP ≥ 0.99 and BS ≥ 95, red color indicates PP ≥ 0.99, and green color indicates BS ≥ 95. The specimen *T. minutisporum* DAOM 107069 was used as the out-group. The strains reported here are highlighted in bold (T = Type specimen).

For sect. *Trichoderma*, 32 isolates were identified. In this case, *T. minutisporum* was used as the out-group. The combined gene matrix formed has 2,976 characters and is subdivided into *rpb2* (728 bp), *act* (681 bp), *its* (544 bp), *tef1α* (605 bp), and *cal* (418 bp). In this analysis, the number of conserved sites, variable sites, and informational sites to parsimony is 1,392, 899, and 697, respectively. The chosen nucleotide substitution models were GTR + I + G for *its*, GTR + I + G for *tef1α*, SYM + G for *rpb2*, SYM + G for *cal*, and GTR + I + G for *act* in the Bayesian inference analysis and GTR + G for maximum likelihood. The species *T. dorothopsis* was reclassified in *T. koningiopsis* due its older name. It was identified in the tree (Figure 5) that the isolates grouped into clades (PP ≥ 0.99 and BT ≥ 95) of *T. asperelloides* (12 isolates), *T. koningiopsis* (9 isolates), *T. asperellum* (4 isolates), *T. hamatum* (2 isolates), *T. atroviride* (1 strain), *T. erinaceum* (1 strain), and *T. subviride* (1 strain). A clade independent of the known species was formed (PP ≥ 0.99 and BT ≥ 95), and this is being proposed as *T. cerradensis.*

### 3.1 Taxonomy

*Trichoderma koningiopsis* Samuels, C. Suarez & H.C. Evans, Studies in Mycology 56: 117. 2006.

*Basionym: Hypocrea koningiopsis* Samuels, Studies in Mycology 56: 117. 2006.

*Synonyms: Trichoderma dorothopsis* A.A. Tomah & J.Z. Zhang, Biological Control 145: 6. 2020.

*Notes:* Considering the available sequences of *T. dorothopsis* (*tef1α*, *rpb2*, and *its*), it positioned within the *T. koningiopsis* clade, showing high support in both the multigene and single-gene analyses of *tef1α* and *rpb2*, meeting the GCPSR criteria. The species *T. dorothopsis* (Tomah et al., 2020) was synonymized under *T. koningiopsis* (Samuels et al., 2006) due its older description.

*Trichoderma lentiforme* (Rehm) P. Chaverri, Samuels & F.B. Rocha, Mycologia 107: 577. 2015.

*Basionym: Hypocrea lentiformis* Rehm, Hedwigia 37: 193. 1898.

*Synonyms: Trichoderma inhamatum* Veerkamp & W. Gams, Caldasia 13: 710. 1983.

*Notes: Trichoderma inhamatum* was synonymized under *T. lentiforme* due to the type of the two species grouping in the same clade of the multi-loci phylogenetic tree. The same results were obtained for the *cal*, *its*, and *rpb2* individual trees (Supplementary Material). In addition, *T. lentiforme* and *T. inhamatum* were morphologically similar and some authors considered these taxa conspecific (Chaverri et al., 2015).

**Figure 6:**
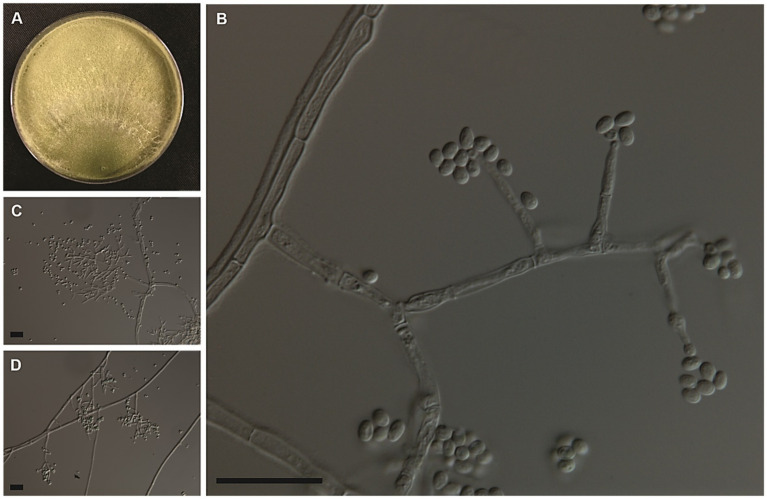
Colony morphologies of the *Trichoderma cerradensis* strain CEN221 formed on PDA after 7 days **(A)**. Morphological structures of *T. cerradensis* formed on PDA after 3 days by microculture technique showing conidiophore, phialides, and globose, subglobose to ovoid conidia **(B–D)**. Bars: 20 μm.

*Trichoderma cerradensis* Peixoto, Pinho, P. Chaverri, & S.C.M. Mello sp. Nov. Figure 6.

MycoBank: 851480.

*Typification*: BRAZIL, Federal District: Brasília, from the soil cultivated with *Gossypium* sp., March 2002, coll. F. G. V. Schmidt (holotype UB24548 permanently preserved in a metabolically inactive state, ex-type living culture CEN221).

*Teleomorph*: Unknown.

*GenBank*: *tef1α* = ON101458; *its* = OM515056; *rpb2* = PQ149277; *cal* = ON241200; and *act =* ON311059.

*Etymology*: The name refers to the Brazilian biome, Cerrado, where this species was isolated.

*Cultural and micrometric characteristics*: Colony radius after 72 h at 25°C on PDA and SNA measuring 63–80 mm, growing more slowly on SNA than on PDA and CMD. The Petri dishes (80 mm Ø) were filled with the CEN221 colony on all culture media after 96 h at 25°C. On PDA, colony radius measured 73–80 mm after 96 h at 30°C. Mycelium cottony with sparse aerial hyphae covering the entire plate, conidia forming abundantly under cottony hyphae after 96 h at 25 and 30°C. No diffusible pigments or distinctive odors observed. On SNA, the colonies measured 77–78 mm in radius after 96 h at 30°C. Colony white with disperse cottony hyphae and dense sporulation at 25°C. Little growth was observed at 15°C and 35°C after 120 h. On CMD, colonies 80 mm in radius after 96 h at 30°C. They were hyaline with disperse cottony hyphae. Masses of green conidia covering the entire plate after 120 h at 25°C. Lowest growth (13–35 mm in radius after 168 h) observed on all culture media at 35°C. Conidiophores pyramidal, with single or opposing branches, terminating in groups of two to three phialides, or solitary. Phialides lageniform, measuring 9.5 ± 1.0 × 3.5 ± 0.5 μm (overall range: 5.5–12.5 × 2.5–4.0 μm), base 1.5–2.5 μm (mean 2.0 μm). Conidial masses olive to green formed by conidia globose, subglobose to ovoid 3.5 ± 0.5 × 3.0 ± 0.5 μm (overall range: 2.5–5.0 × 2.0–4.0 μm) (Table 3). Chlamydospores not observed.

**Table 3:** Morphological characteristics of *Trichoderma cerradensis* sp. nov. compared to closely related strains of *Trichoderma* species.

Espécie	Phialides		Conidia		Chlamydospore
	LxW (μm)	Shape	LxW (μm)	Shape	
*T. cerradensis*^1^ sp. nov.	5.5–12.5 × 2.0–4.0	Lageniform	2.5–5.0 × 2.0–4.0	Globose, subglobose to ovoid, olive green	Not observed
*T. gamsii* ^2^	5.2–18.5 × 1,5–4.0	Lageniform	3.2–5.8 × 2.2–3.2	Ellipsoidal, oblong to ovoid	Often, terminal
*T. hispanicum* ^3^	4.5–17.0 × 2.0–4.0	Lageniform or ampuliform	3.5–6.0 × 2.8–4.0	Oblong to elipsoidal, green	Not often, terminal
*T. neokoningii* ^2^	5.0–10.0 × 2.2–3.0	Lageniform	3.5–4.0 × 2.5–3.0	Ellipsoidal to oblong	Abundant, terminal
*T. samuelsii* ^3^	5.0–16.0 × 2.2–3.5	Lagerniform	3.7–5.7 × 2.4–3.7	Elipsoidal, green	Not often, terminal and intercalate

^1This study; 2Jaklitsch et al., 2006; 3Jaklitsch et al., 2012.^

*Additional specimen examined*: BRAZIL, Federal District: *Rural Nucleus of “Rajadinha,”* Brasília, from soil cultivated with *Cucurbita pepo*, February 2012, coll. J.B.T. da Silva (culture CEN1348).

*Known substrate*: Soil under *Gossypium* sp. and *Cucurbita pepo*.

*Known geographic distribution*: Brasília, Brazil.

*Notes*: Considering the GCPSR criteria, the new species was assigned, where in all individual trees, the *T. cerradensis* clade was highlighted with high support in ML and BI, thus meeting the criteria. *Trichoderma cerradensis* was closely related to *T. gamsii*, *T. hispanicum*, *T. neokoningii*, and *T. samuelsii*. Although there is an overlap in phialides and conidial measures, in comparison with *T. gamsii*, *T. hispanicum*, and *T. samuelsii*, phialides of *T. cerradensis* are shorter. *Trichoderma cerradensis* conidia were globose to ovoid, while *T. gamsii*, *T. hispanicum*, *T. neokoningii* and *T. samuelsii* conidia were ellipsoidal, oblong, and sometimes ovoid. Chlamydospores were not observed in *Trichoderma cerradensis* sp. nov. In contrast, *T. gamsii* and *T. neokoningii* often produced terminal chlamydospores, *T. hispanicum* sometimes produced terminal, and *T. samuelsii* exhibited terminal and intercalate chlamydospores. *Trichoderma cerradensis* sp. nov. was distinguished from all other *Trichoderma* species and well supported in the phylogenetic analyses (Figure 5).

## 4 Discussion

The present study demonstrates that within a relatively small collection of *Trichoderma* isolates for biological control, there are more species than previously reported. Many biocontrol isolates were typically classified in the *T. harzianum* or *T. atroviride* species complexes (Jaklitsch et al., 2006; 2012). This study identified 19 species of *Trichoderma*, including the new species *T. cerradensis.* In addition to *T. cerradensis*, some of the other species identified were *T. afarasin*, *T. asperelloides*, *T. asperellum*, *T. atroviride*, *T. austroindianum*, *T. azevedoi*, *T. brevicompactum*, *T. erinaceum*, *T. ghanense*, *T. hamatum*, *T. hortense*, *T. koningiopsis*, *T. lentiforme*, *T. longibrachiatum*, *T. peberdyi*, *T. rifaii*, *T. spirale*, and *T. subviride.*

*Trichoderma austroindianum* and *T. hortense* were recently identified from Argentinian soils (Barrera et al., 2021). *Trichoderma brevicompactum* has been reported in Brazil as an endophyte in healthy *Theobroma cacao* tissues (Novais Bastos, 2012), unlike the specimens recovered from soils in this study (Table 1). *Trichoderma asperellum*, *T. koningiopsis*, and *T. erinaceum* were identified from soil samples under common bean (*Phaseolus vulgaris*) crops and rubber trees native to the Brazilian Amazon (Lopes et al., 2012; Brito et al., 2023). The isolates characterized in the present study were from natural habitats in the Cerrado (i.e., *T. asperellum* and *T. koningiopsis*), Amazon Forest (i.e., *T. koningiopsis*), and Pantanal of Mato Grosso (i.e., *T. erinaceum*). Therefore, the data obtained in the present study suggest the versatility of *Trichoderma* in terms of its ability to colonize different substrata, habitats, and regions.

The data obtained here also provide additional records for species, e.g., *T. longibrachiatum* from Federal District and Mato Grosso State and *T. hamatum* from Federal District, that were reported in soil contaminated with textile laundry discharge in Pernambuco and from mangrove sediment in Bahia (da Silva et al., 2016). *Trichoderma atroviride* (from Goiás State) was previously reported from a soil sample from the Amazon Forest (Grigorevski-Lima et al., 2013) and *T. spirale* (from Federal District) in samples from a forest agroecosystem in Bahia (Reis et al., 2015) and Brazilian Amazon (Brito et al., 2023). *Trichoderma lentiforme* (São Paulo State and Federal District) has already been found colonizing palm leaves in Santa Catarina, rubber trees native in Brazilian Amazon, and as an endophyte of several tropical trees (Chaverri et al., 2015; Brito et al., 2023). *Trichoderma afroharzianum* (Federal District, Goiás, Mato Grosso and São Paulo) is widely distributed and was even found colonizing fungal gardens of the leaf-cutting ant *Atta sexdens* in São Paulo (Montoya et al., 2016). Inglis et al. (2020) described two new species, namely, *T. azevedoi* and *T. peberdyi*, from soil samples of *Allium sativum* and *A. cepa* collected from a different area in Brazil (Inglis et al., 2020). It is interesting to mention that all these isolates belong to the same EMBRAPA Culture Collection. In addition, the *T. afroharzianum* isolate CEN287 is the active ingredient of Habitat®, a commercial biological control product used against *Sclerotinia sclerotiorum* and *Rhizoctonia solani*.

Until the present study, *T. rifaii* had only been found as an endophyte in leaves and stems of tropical trees (*Theobroma cacao* and *T. gileri*) from Ecuador (Chaverri et al., 2015). Interestingly, the results obtained here show the presence of this species recovered from samples of native and cultivated soil (different crops) from the Federal District, from soil under cultivated rice and sorghum in Goiás State, and soil from the Pantanal in Mato Grosso State (Table 1). However, this is the first report of this species in Brazil. According to Chaverri et al. (2015), *T. rifaii* has *its* sequences identical to *T. endophyticum*, which explains the proximity of the clades that define these species.

According to a survey carried out by Menolli and Sánchez-García (2020), there are currently 289 *its* sequences of *Trichoderma* isolates from Brazil in GenBank. In addition, according to these authors, many sequences in GenBank do not have information regarding the country of origin in their metadata; therefore, the number of *Trichoderma* species in Brazil may be greatly underestimated.

The present study not only demonstrates the usefulness of fungal culture collections but also increases the cataloging and correct identification of fungal biodiversity that may be applied in organic or sustainable agriculture. In addition, the present study confirms that *its* is useless in accurate identification of *Trichoderma* and that, in contrast, *tef1α* and *rpb2* continue to be useful as secondary barcodes and for biodiversity discovery (Chaverri and Samuels, 2003; Chaverri et al., 2015; Cai and Druzhinina, 2021).

## Data Availability

The datasets presented in this study can be found in online repositories. The names of the repository/repositories and accession number(s) can be found in the article/Supplementary material.

## Footnotes

^1^ https://www.dna-dragon.com

^2^ https://www.ncbi.nlm.nih.gov/genbank
