# Supplementary material for: Trichoderma collection from Brazilian soil reveals a new species: T. cerradensis Sp. nov
Source: Front Microbiol. 2025 Feb 11;16:1279142. doi: 10.3389/fmicb.2025.1279142 (PMC11850384; doi:10.3389/fmicb.2025.1279142)

A Brazilian soil *Trichoderma* collection reveals a new species, *T. cerradensis* sp. nov.

**Supplementary Material 1.** Bayesian phylogenetic tree based on TEF1α sequences of Sect. *Trichoderma* complex. Bayesian posterior probability values are indicated at the nodes, and the scale bar represents the number of expected changes per site. The specimen *Trichoderma* *minutisporum* DAOM 107069^T^ was used as outgroup. (^T^ =Type specimen)


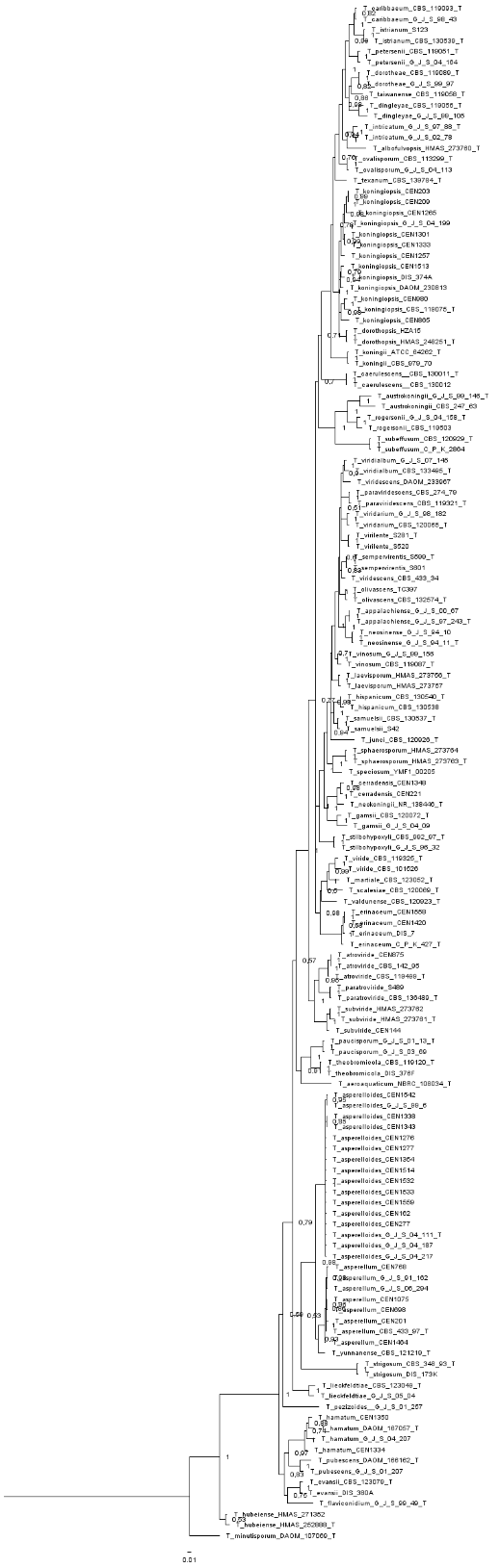


**Supplementary Material 2.** Bayesian phylogenetic tree based on RPB2 sequences of Sect. *Trichoderma* complex. Bayesian posterior probability values are indicated at the nodes, and the scale bar represents the number of expected changes per site. The specimen *Trichoderma* *minutisporum* DAOM 107069^T^ was used as outgroup. (^T^ =Type specimen)


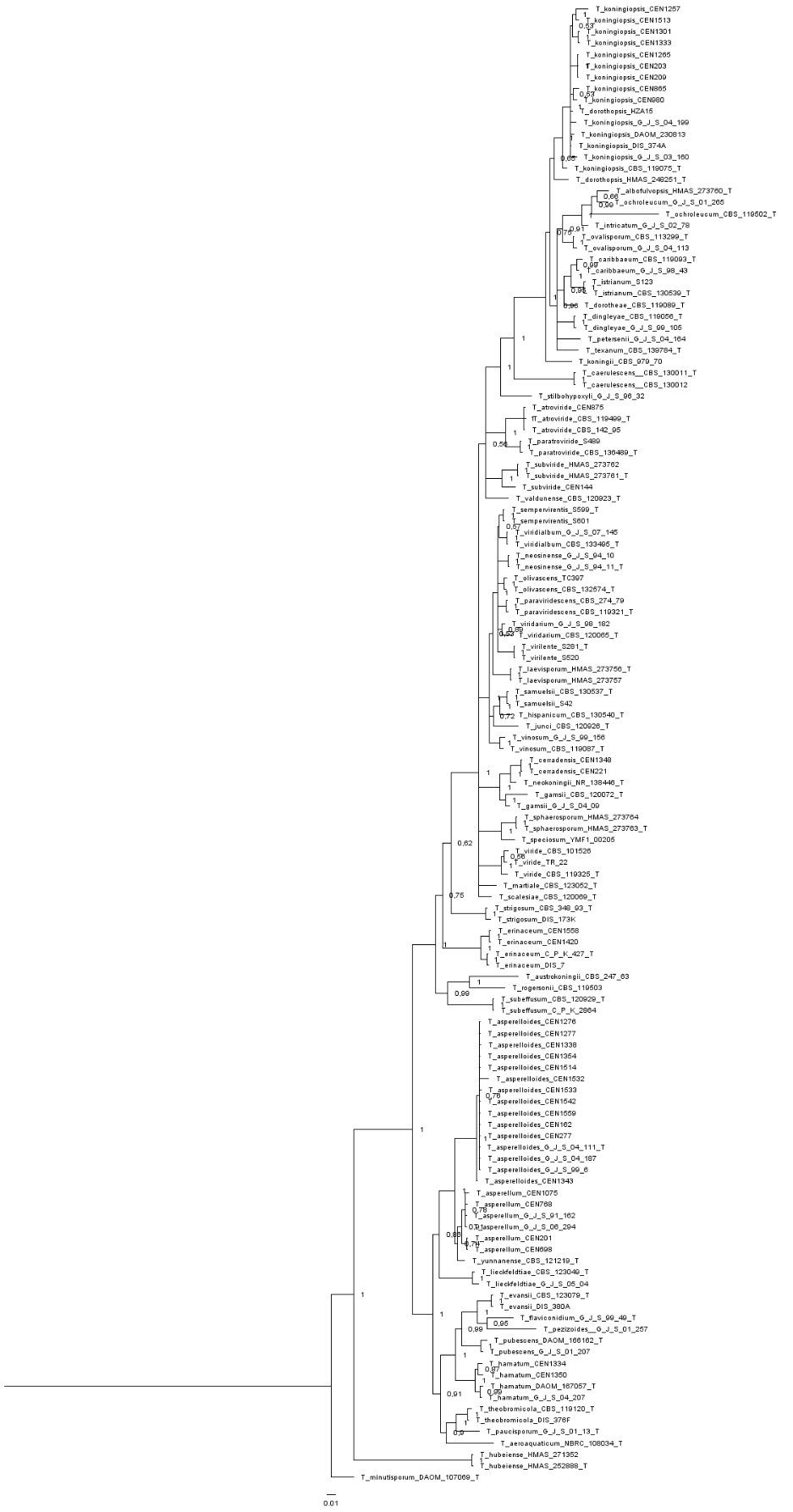


**Supplementary Material 3.** Bayesian phylogenetic tree based on ITS sequences of Sect. *Trichoderma* complex. Bayesian posterior probability values are indicated at the nodes, and the scale bar represents the number of expected changes per site. The specimen *Trichoderma* *minutisporum* DAOM 107069^T^ was used as outgroup. (^T^ =Type specimen)


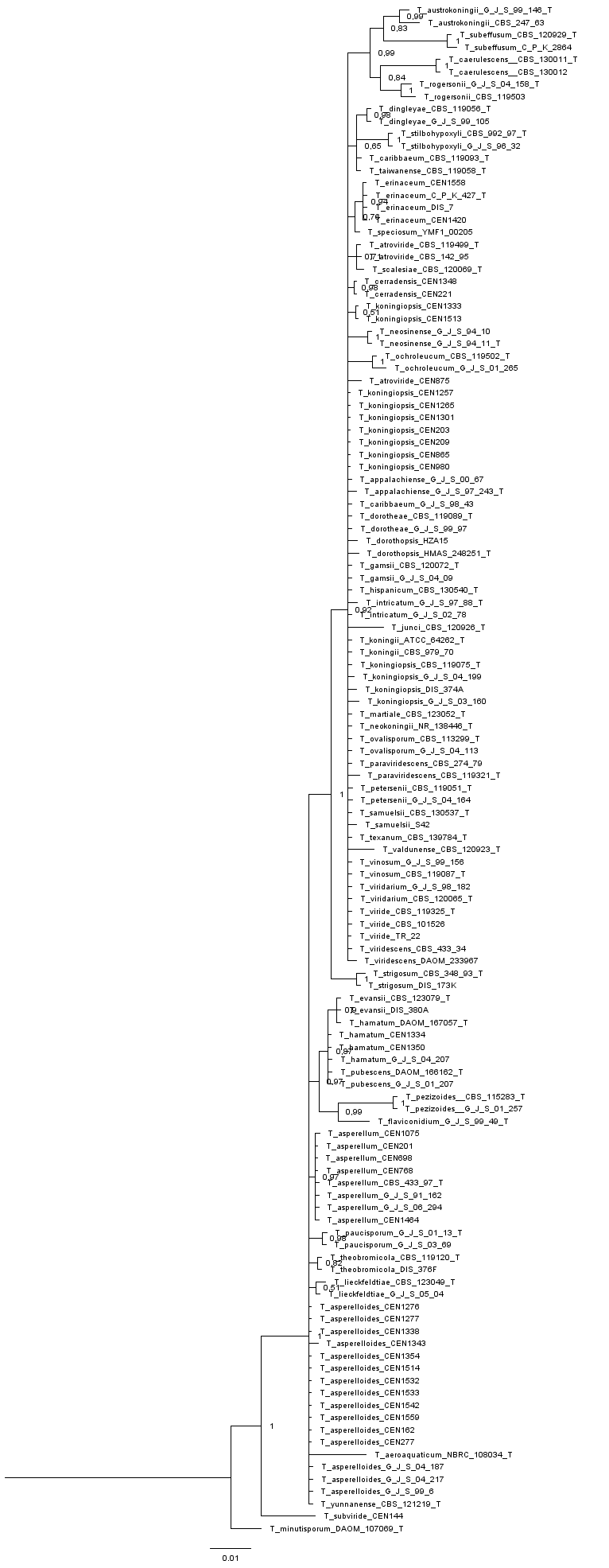


**Supplementary Material 4.** Bayesian phylogenetic tree based on CAL sequences of Sect. *Trichoderma* complex. Bayesian posterior probability values are indicated at the nodes, and the scale bar represents the number of expected changes per site. The specimen *Trichoderma* *minutisporum* DAOM 107069^T^ was used as outgroup. (^T^ =Type specimen)


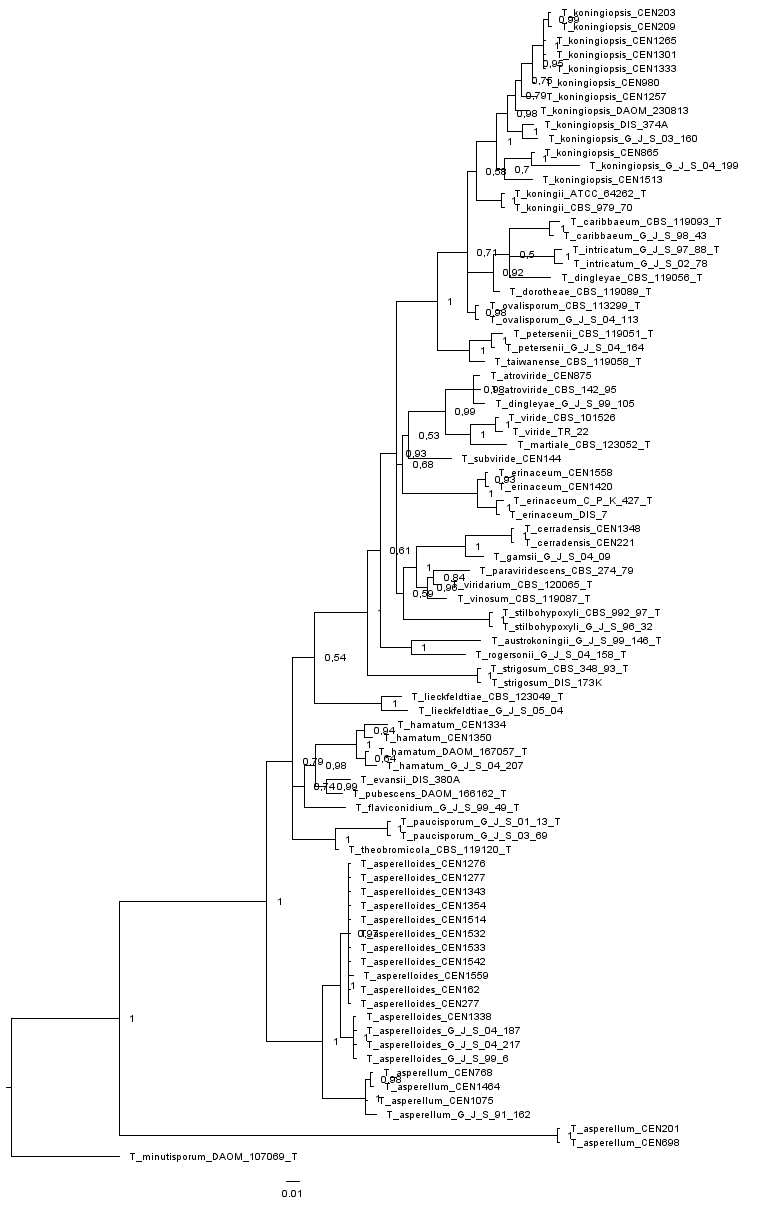


**Supplementary Material 5.** Bayesian phylogenetic tree based on ACT sequences of Sect. *Trichoderma* complex. Bayesian posterior probability values are indicated at the nodes, and the scale bar represents the number of expected changes per site. The specimen *Trichoderma* *minutisporum* DAOM 107069^T^ was used as outgroup. (^T^ =Type specimen)


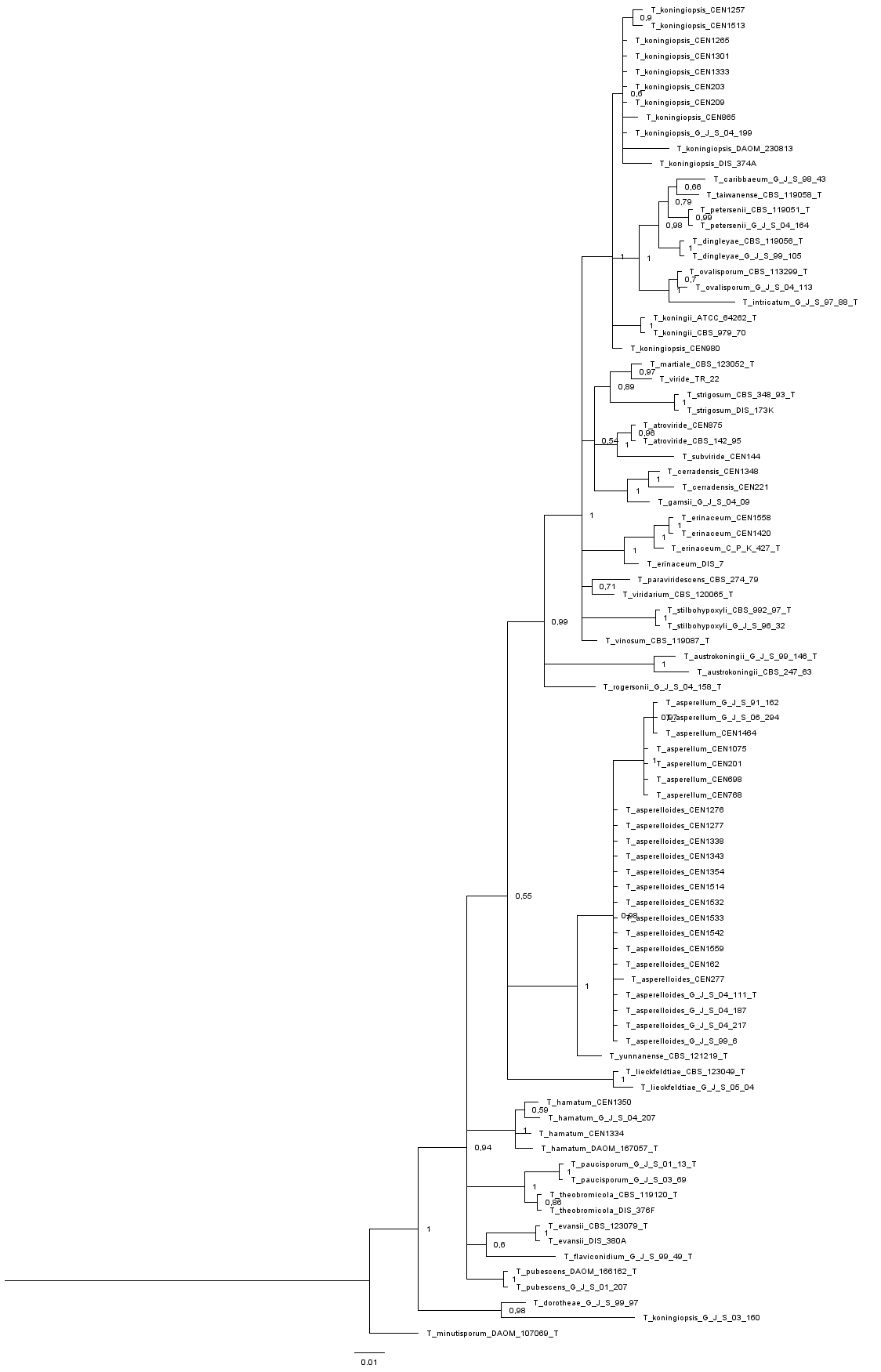


**Supplementary Material 6.** Bayesian phylogenetic tree based on TEF1α sequences of *Harzianum* complex. Bayesian posterior probability values are indicated at the nodes, and the scale bar represents the number of expected changes per site. The specimen *Trichoderma* *viride* CBS 101526^T^ was used as outgroup. (^T^ =Type specimen)


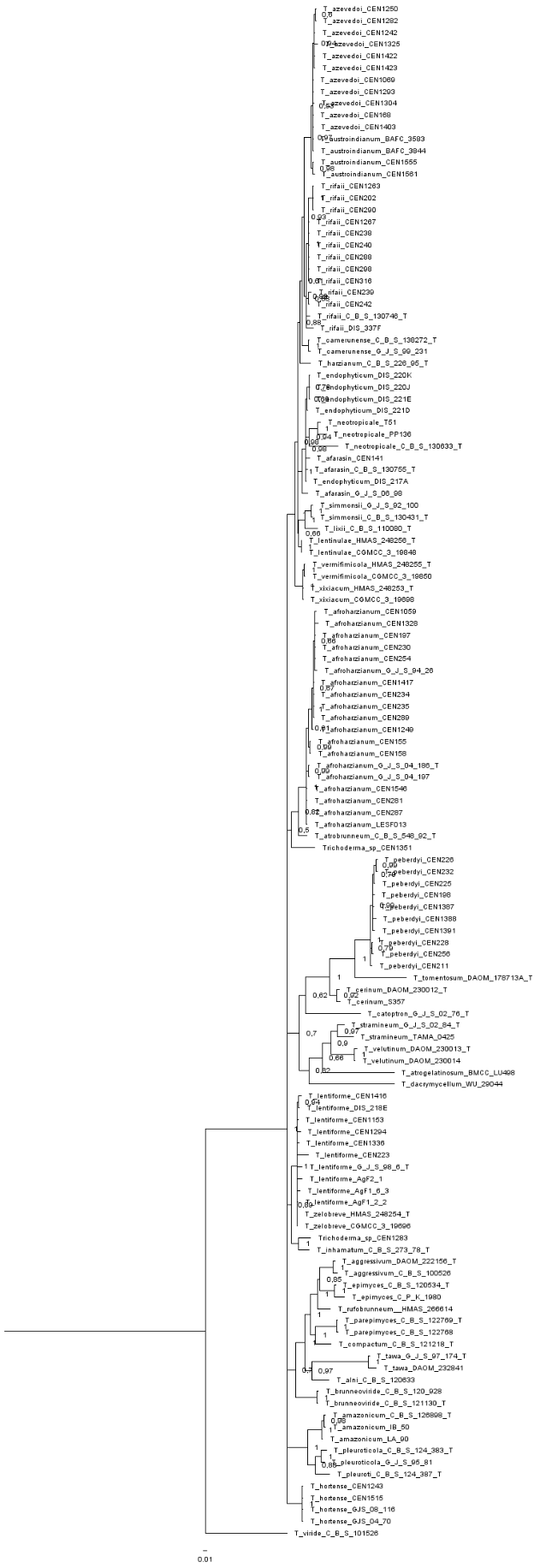


**Supplementary Material 7.** Bayesian phylogenetic tree based on RPB2 sequences of *Harzianum* complex. Bayesian posterior probability values are indicated at the nodes, and the scale bar represents the number of expected changes per site. The specimen *Trichoderma* *viride* CBS 101526^T^ was used as outgroup. (^T^ =Type specimen)


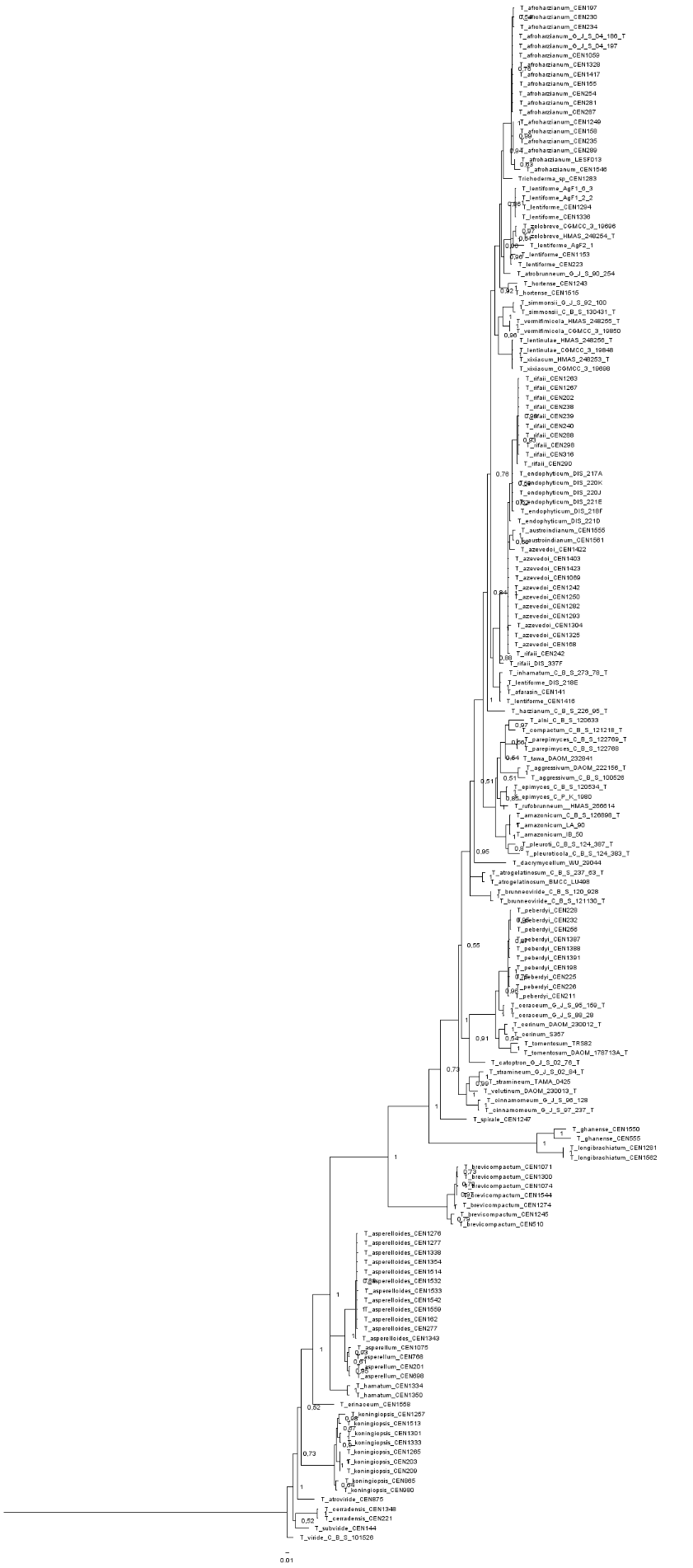


**Supplementary Material 8.** Bayesian phylogenetic tree based on ITS sequences of *Harzianum* complex. Bayesian posterior probability values are indicated at the nodes, and the scale bar represents the number of expected changes per site. The specimen *Trichoderma* *viride* CBS 101526^T^ was used as outgroup. (^T^ =Type specimen)


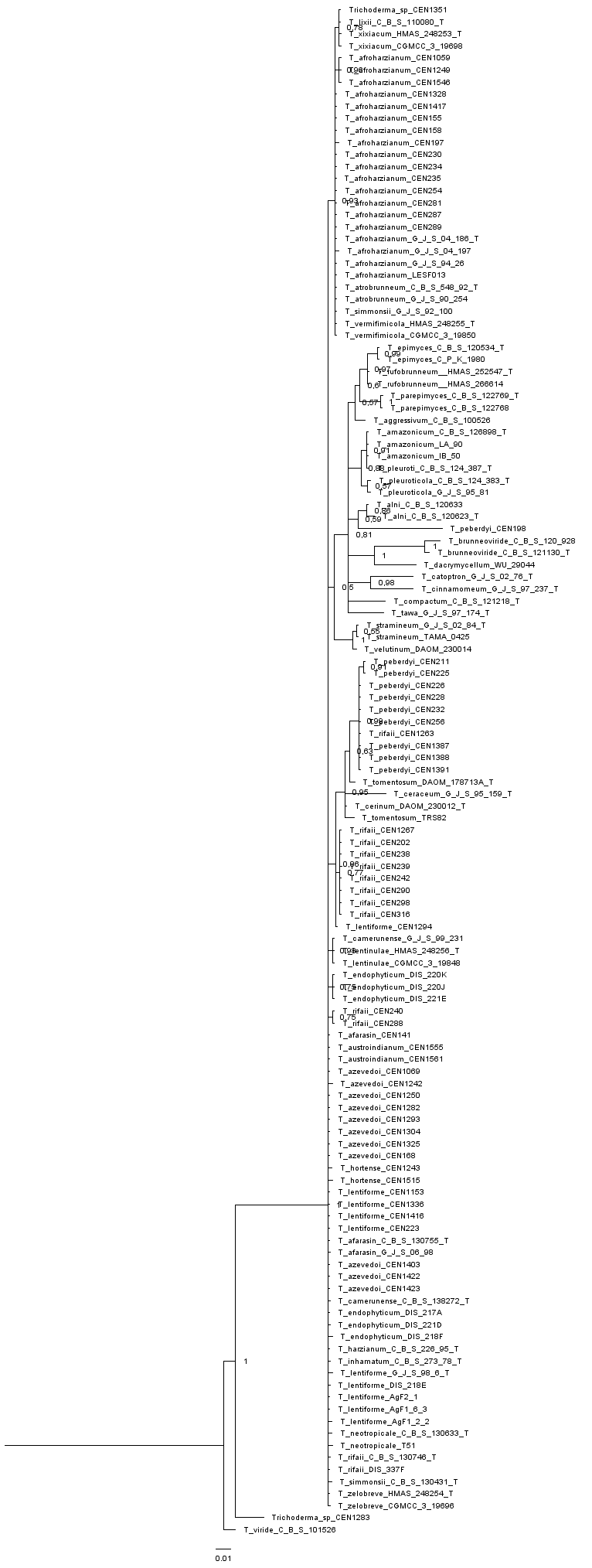


**Supplementary Material 9.** Bayesian phylogenetic tree based on CAL sequences of *Harzianum* complex. Bayesian posterior probability values are indicated at the nodes, and the scale bar represents the number of expected changes per site. The specimen *Trichoderma* *viride* CBS 101526^T^ was used as outgroup. (^T^ =Type specimen)


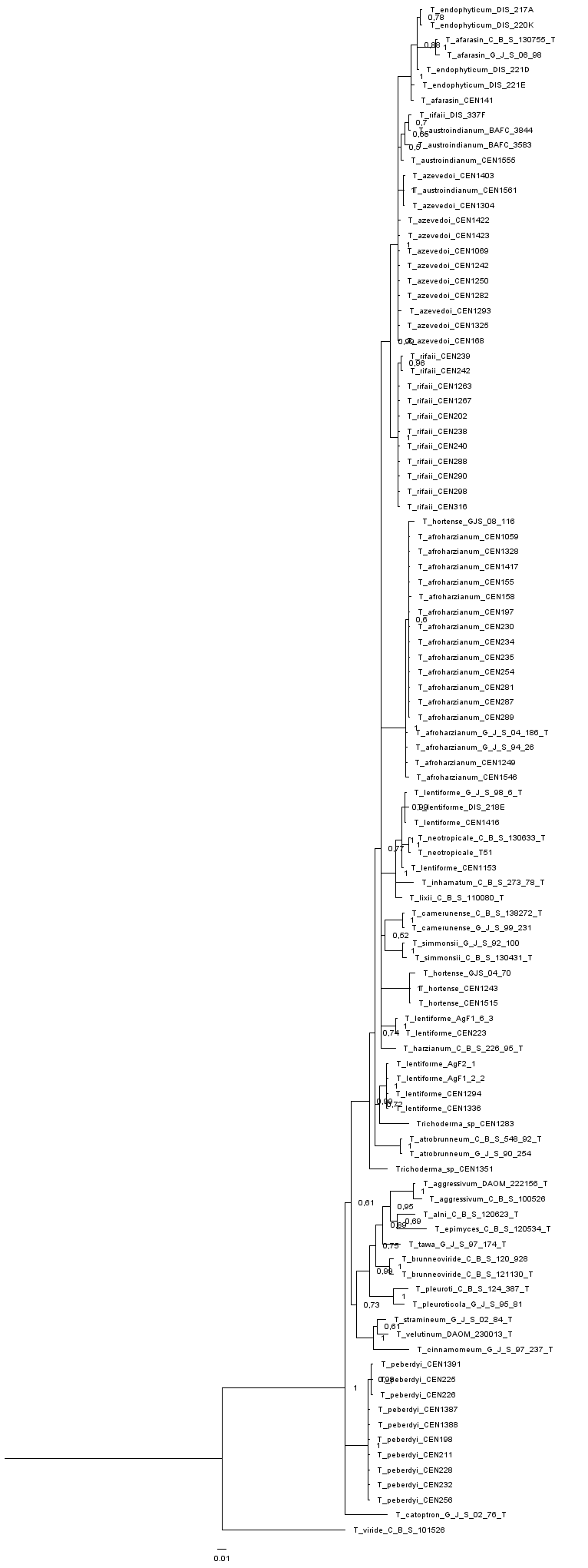


**Supplementary Material 10.** Bayesian phylogenetic tree based on ACT sequences of *Harzianum* complex. Bayesian posterior probability values are indicated at the nodes, and the scale bar represents the number of expected changes per site. (^T^ =Type specimen)


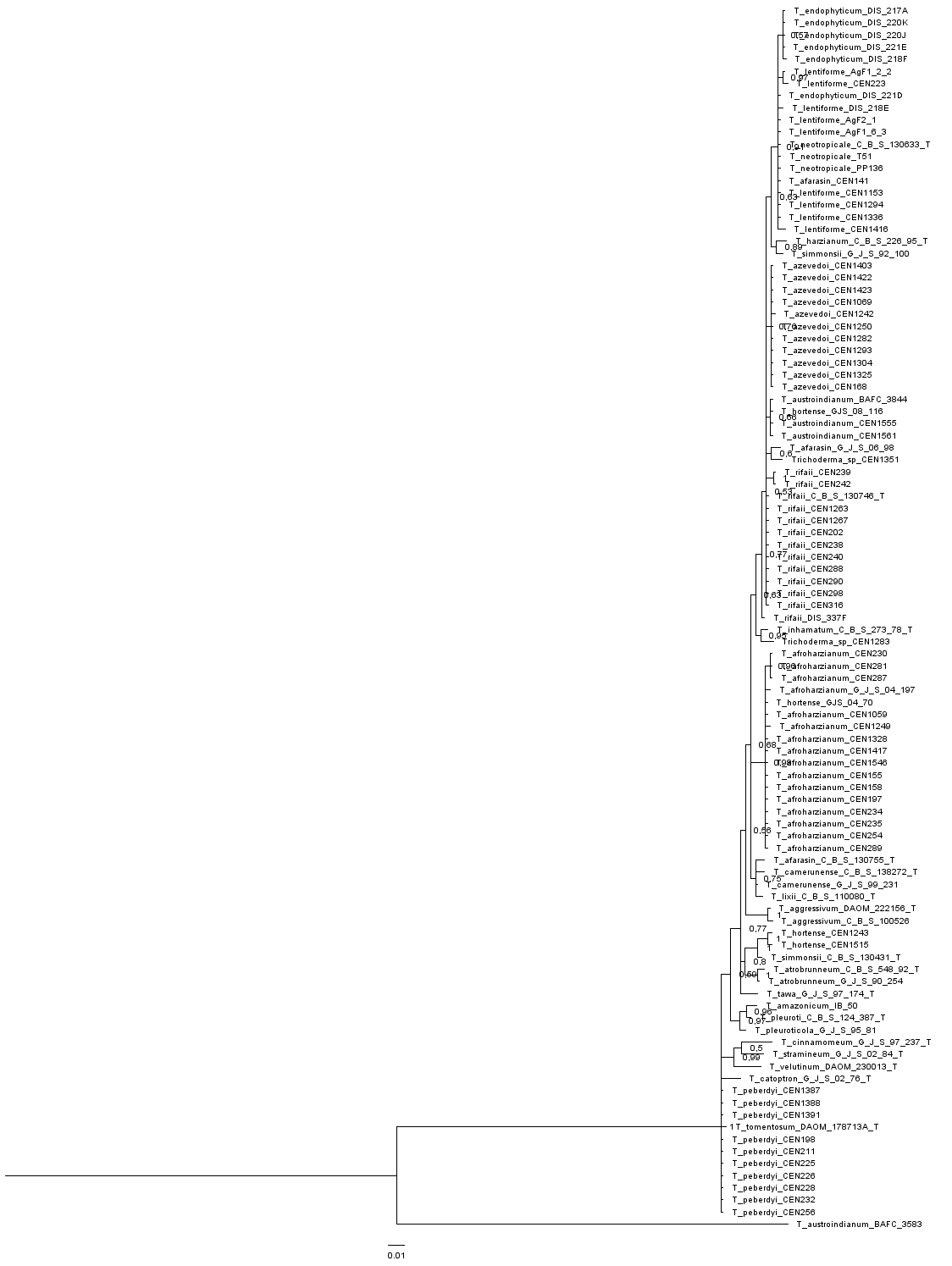


**Supplementary Material 11.** Bayesian phylogenetic tree based on TEF1α sequences of *Brevicompactum* complex. Bayesian posterior probability values are indicated at the nodes, and the scale bar represents the number of expected changes per site. The specimen *Trichoderma* *minutisporum* DAOM 167069^T^ was used as outgroup. (^T^ =Type specimen)

**
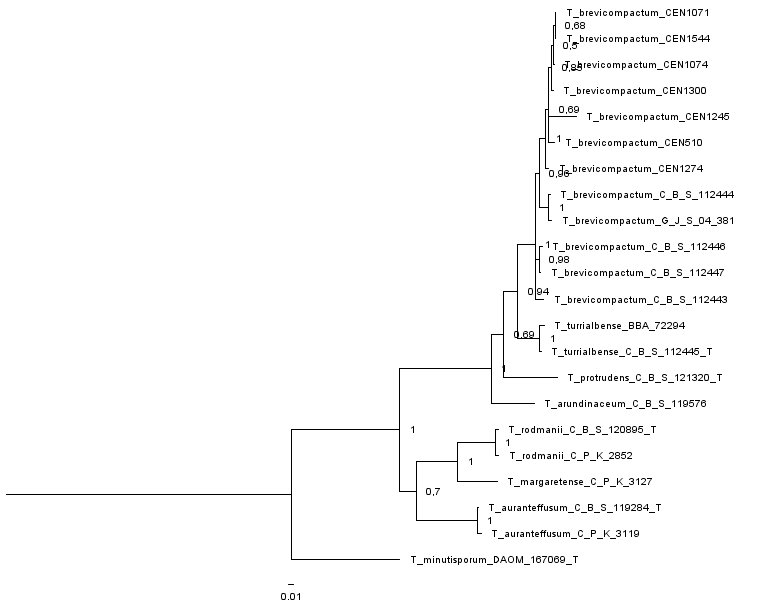
**

**Supplementary Material 12.** Bayesian phylogenetic tree based on RPB2 sequences of *Brevicompactum* complex. Bayesian posterior probability values are indicated at the nodes, and the scale bar represents the number of expected changes per site. The specimen *Trichoderma* *minutisporum* DAOM 167069^T^ was used as outgroup. (^T^ =Type specimen)

**
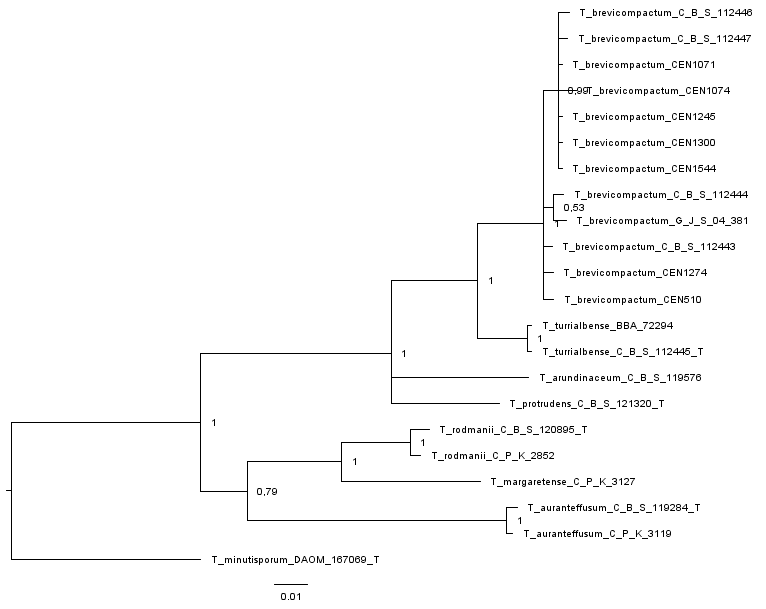
**

**Supplementary Material 13.** Bayesian phylogenetic tree based on ITS sequences of *Brevicompactum* complex. Bayesian posterior probability values are indicated at the nodes, and the scale bar represents the number of expected changes per site. The specimen *Trichoderma* *minutisporum* DAOM 167069^T^ was used as outgroup. (^T^ =Type specimen)

**
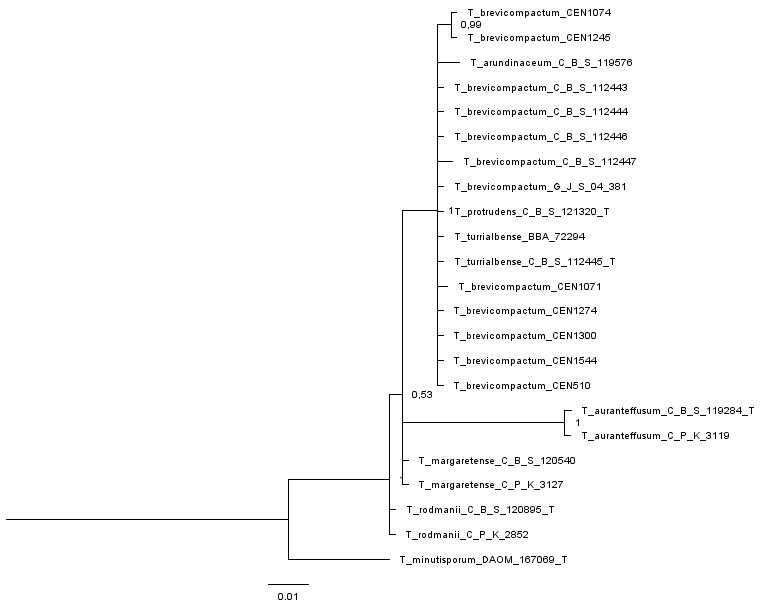
**

**Supplementary Material 14.** Bayesian phylogenetic tree based on CAL sequences of *Brevicompactum* complex. Bayesian posterior probability values are indicated at the nodes, and the scale bar represents the number of expected changes per site. The specimen *Trichoderma* *minutisporum* DAOM 167069^T^ was used as outgroup. (^T^ =Type specimen)

**
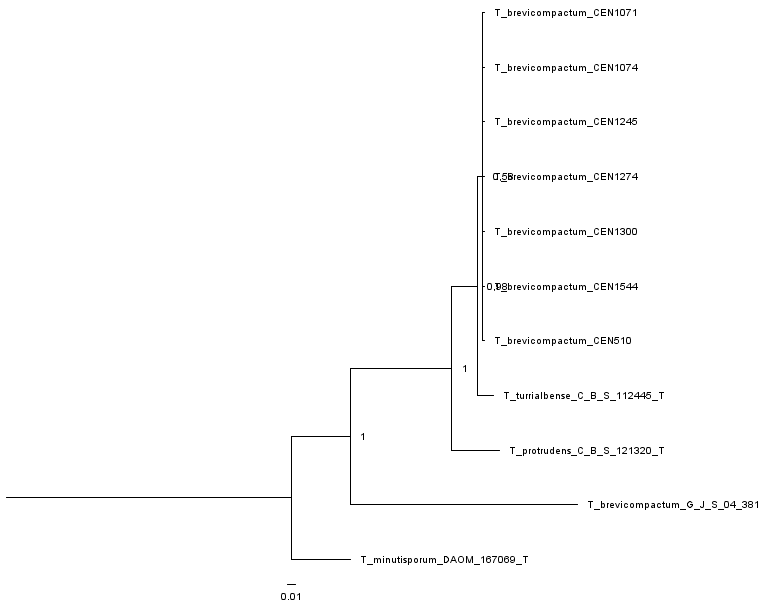
**

**Supplementary Material 15.** Bayesian phylogenetic tree based on ACT sequences of *Brevicompactum* complex. Bayesian posterior probability values are indicated at the nodes, and the scale bar represents the number of expected changes per site. The specimen *Trichoderma* *minutisporum* DAOM 167069^T^ was used as outgroup. (^T^ =Type specimen)

**
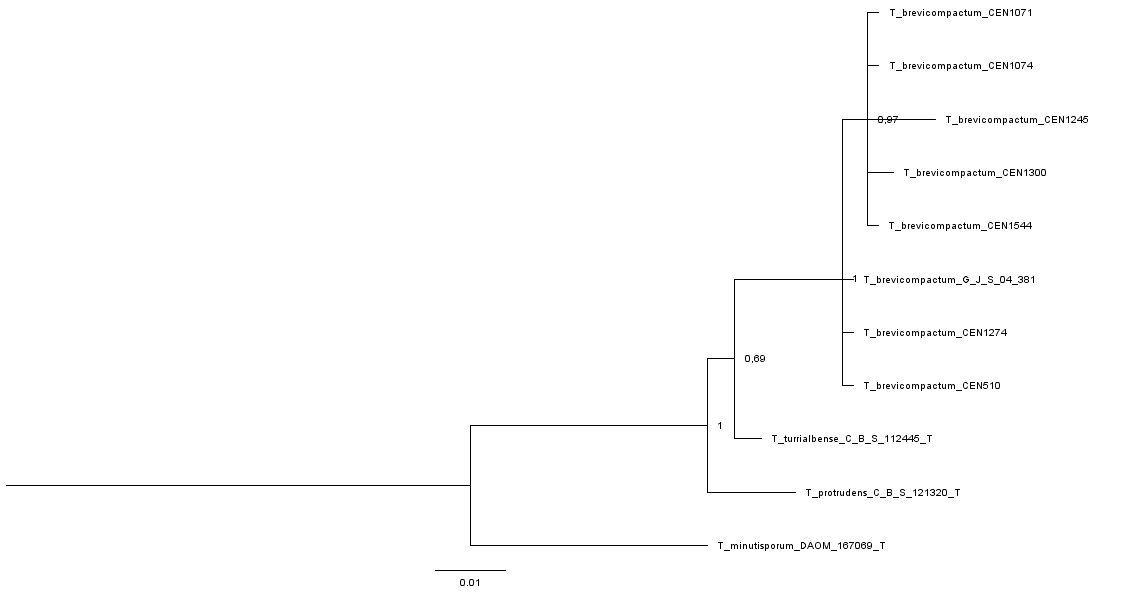
**

**Supplementary Material 16.** Bayesian phylogenetic tree based on TEF1α sequences of *Longibrachiatum* complex. Bayesian posterior probability values are indicated at the nodes, and the scale bar represents the number of expected changes per site. The specimen *Trichoderma* *minutisporum* DAOM 167069^T^ was used as outgroup. (^T^ =Type specimen)

**
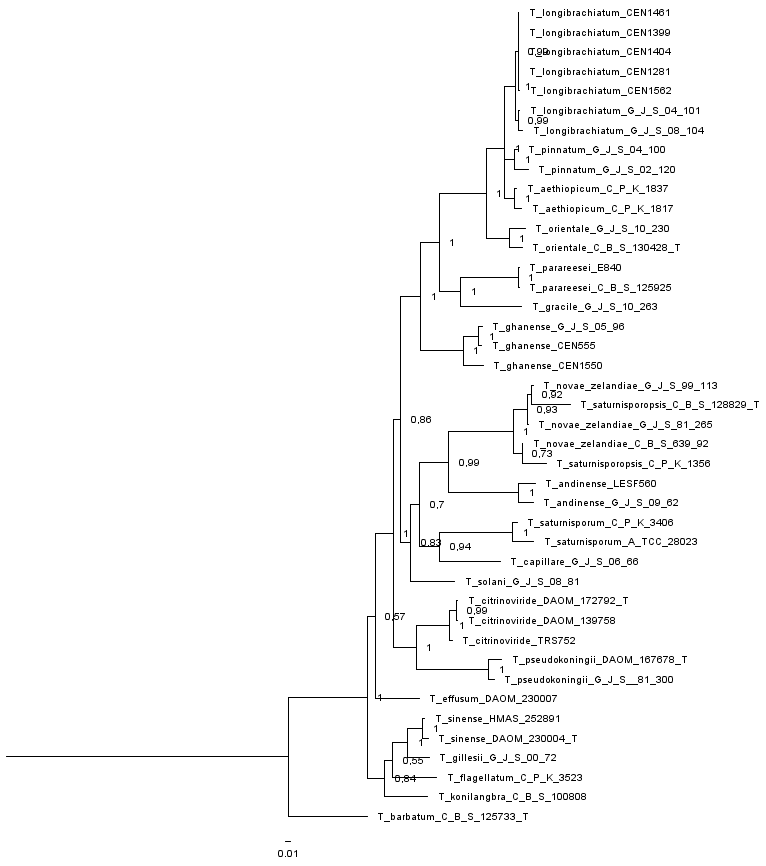
**

**Supplementary Material 17.** Bayesian phylogenetic tree based on RPB2 sequences of *Longibrachiatum* complex. Bayesian posterior probability values are indicated at the nodes, and the scale bar represents the number of expected changes per site. The specimen *Trichoderma* *minutisporum* DAOM 167069^T^ was used as outgroup. (^T^ =Type specimen)

**
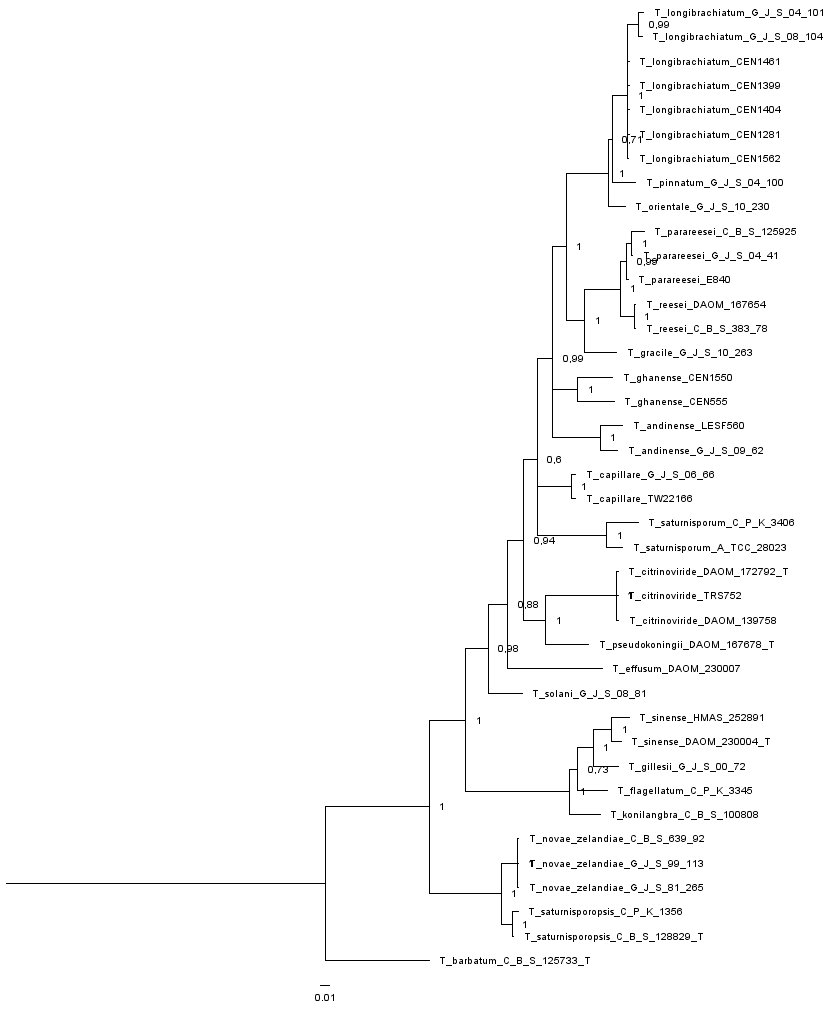
**

**Supplementary Material 18.** Bayesian phylogenetic tree based on ITS sequences of *Longibrachiatum* complex. Bayesian posterior probability values are indicated at the nodes, and the scale bar represents the number of expected changes per site. The specimen *Trichoderma* *minutisporum* DAOM 167069^T^ was used as outgroup. (^T^ =Type specimen)

**
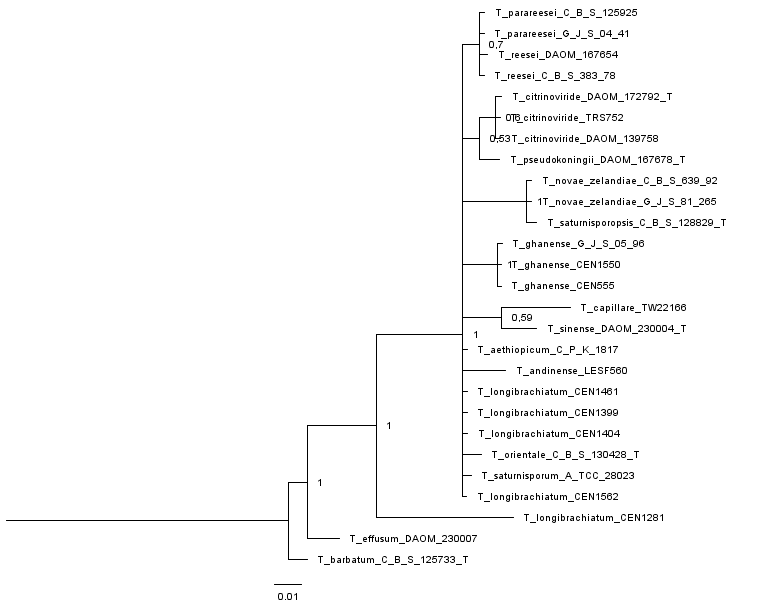
**

**Supplementary Material 19.** Bayesian phylogenetic tree based on CAL sequences of *Longibrachiatum* complex. Bayesian posterior probability values are indicated at the nodes, and the scale bar represents the number of expected changes per site. The specimen *Trichoderma* *minutisporum* DAOM 167069^T^ was used as outgroup. (^T^ =Type specimen)

**
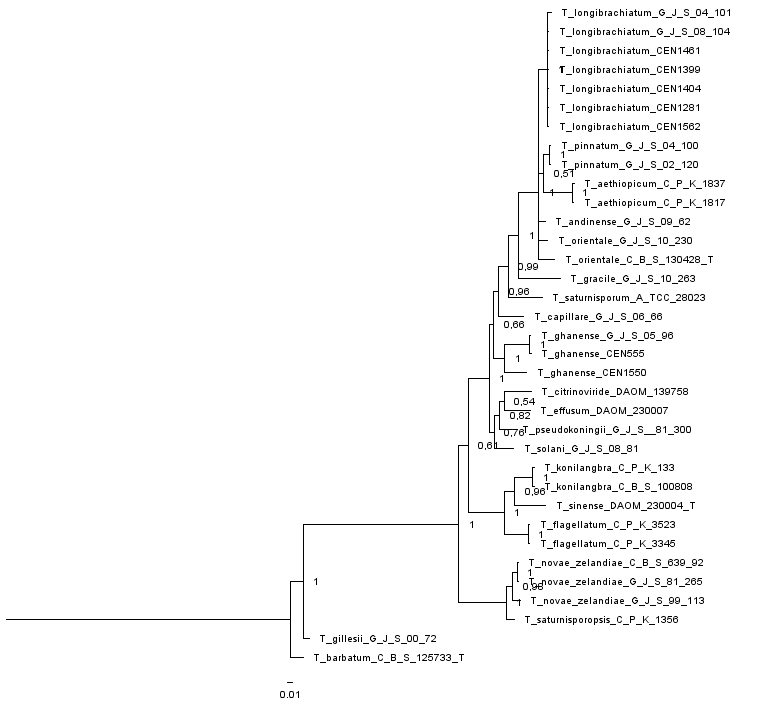
**

**Supplementary Material 20.** Bayesian phylogenetic tree based on ACT sequences of *Longibrachiatum* complex. Bayesian posterior probability values are indicated at the nodes, and the scale bar represents the number of expected changes per site. (^T^ =Type specimen)

**
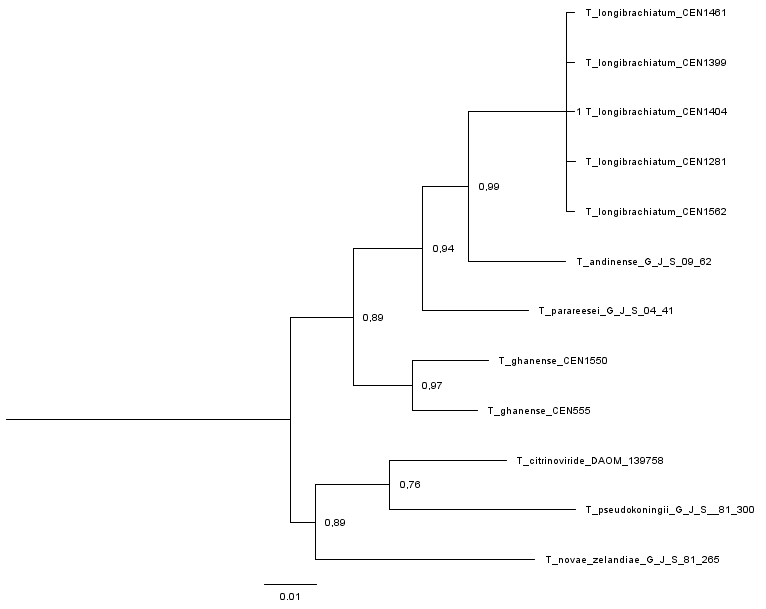
**

**Supplementary Material 21.** Bayesian phylogenetic tree based on TEF1α sequences of *Strictipilosa* and *Stromaticum* complex. Bayesian posterior probability values are indicated at the nodes, and the scale bar represents the number of expected changes per site. The specimen *Trichoderma* *semiorbis* G.J.S. 99.108^T^ was used as outgroup. (^T^ =Type specimen)

**
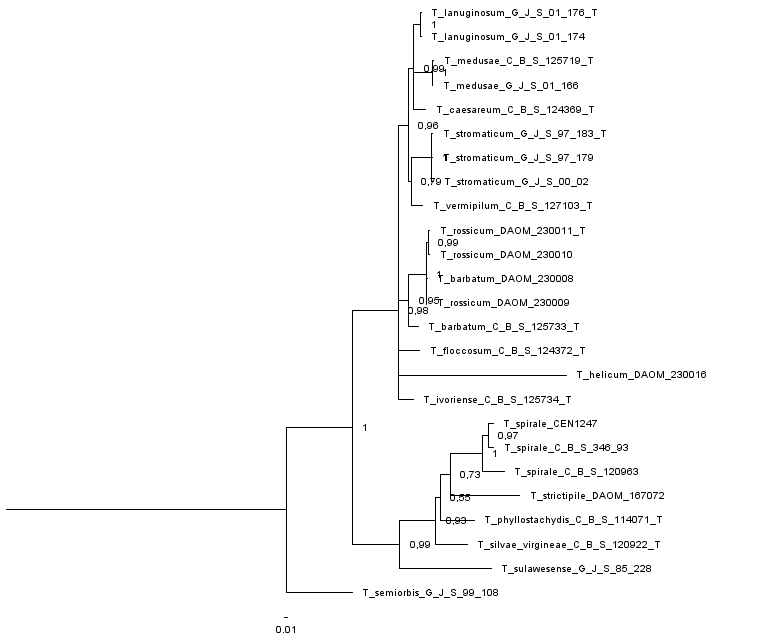
**

**Supplementary Material 22.** Bayesian phylogenetic tree based on RPB2 sequences of *Strictipilosa* and *Stromaticum* complex. Bayesian posterior probability values are indicated at the nodes, and the scale bar represents the number of expected changes per site. The specimen *Trichoderma* *semiorbis* G.J.S. 99.108^T^ was used as outgroup. (^T^ =Type specimen)

**
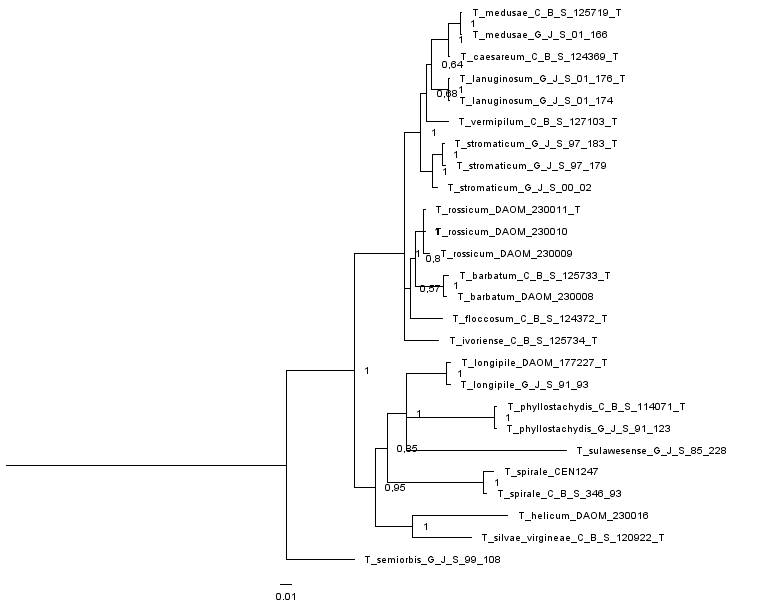
**

**Supplementary Material 23.** Bayesian phylogenetic tree based on ITS sequences of *Strictipilosa* and *Stromaticum* complex. Bayesian posterior probability values are indicated at the nodes, and the scale bar represents the number of expected changes per site. The specimen *Trichoderma* *semiorbis* G.J.S. 99.108^T^ was used as outgroup. (^T^ =Type specimen)

**
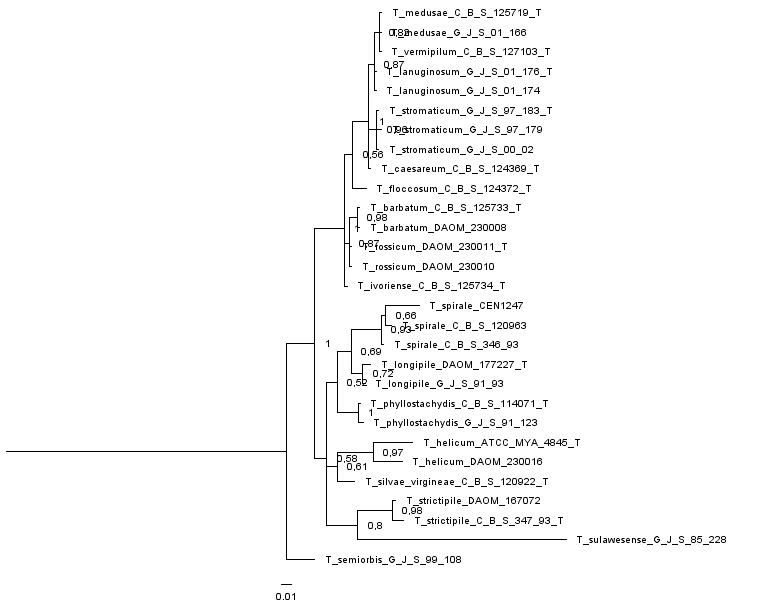
**

**Supplementary Material 24.** Bayesian phylogenetic tree based on CAL sequences of *Strictipilosa* and *Stromaticum* complex. Bayesian posterior probability values are indicated at the nodes, and the scale bar represents the number of expected changes per site. The *Trichoderma* *semiorbis* G.J.S. 99.108^T^ was used as outgroup. (^T^ =Type specimen)

**
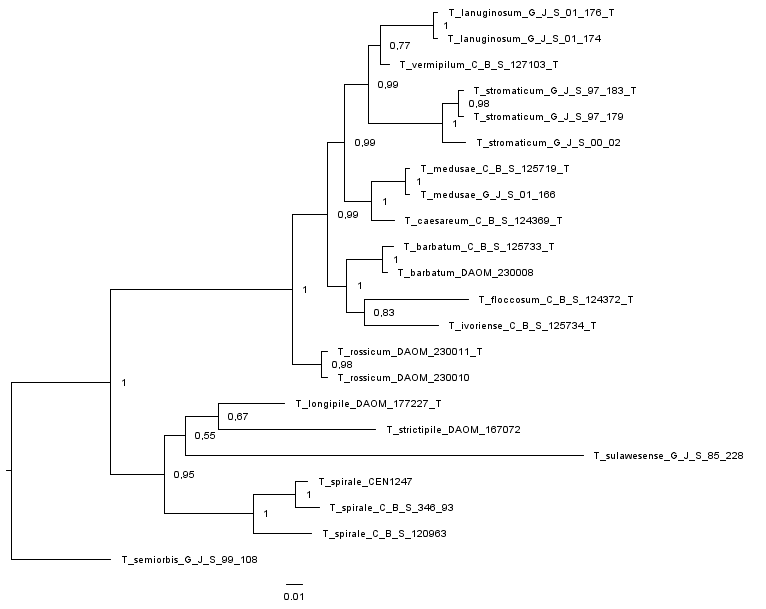
**

**Supplementary Material 25.** Bayesian phylogenetic tree based on ACT sequences of *Strictipilosa* and *Stromaticum* complex. Bayesian posterior probability values are indicated at the nodes, and the scale bar represents the number of expected changes per site. The specimen *Trichoderma* *semiorbis* G.J.S. 99.108^T^ was used as outgroup. (^T^ =Type specimen)
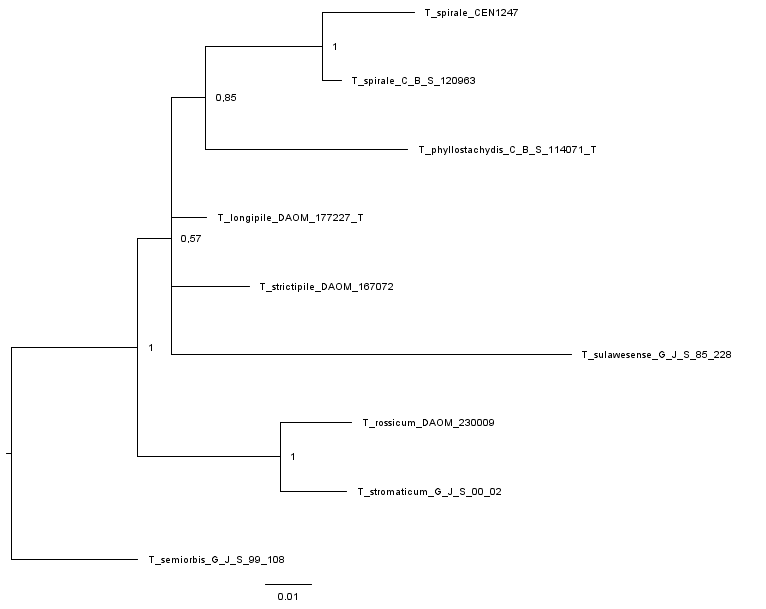


**Supplementary Material 26.** Bayesian phylogenetic tree based on TEF1α sequences of all strains of this study. Bayesian posterior probability values are indicated at the nodes, and the scale bar represents the number of expected changes per site.

**
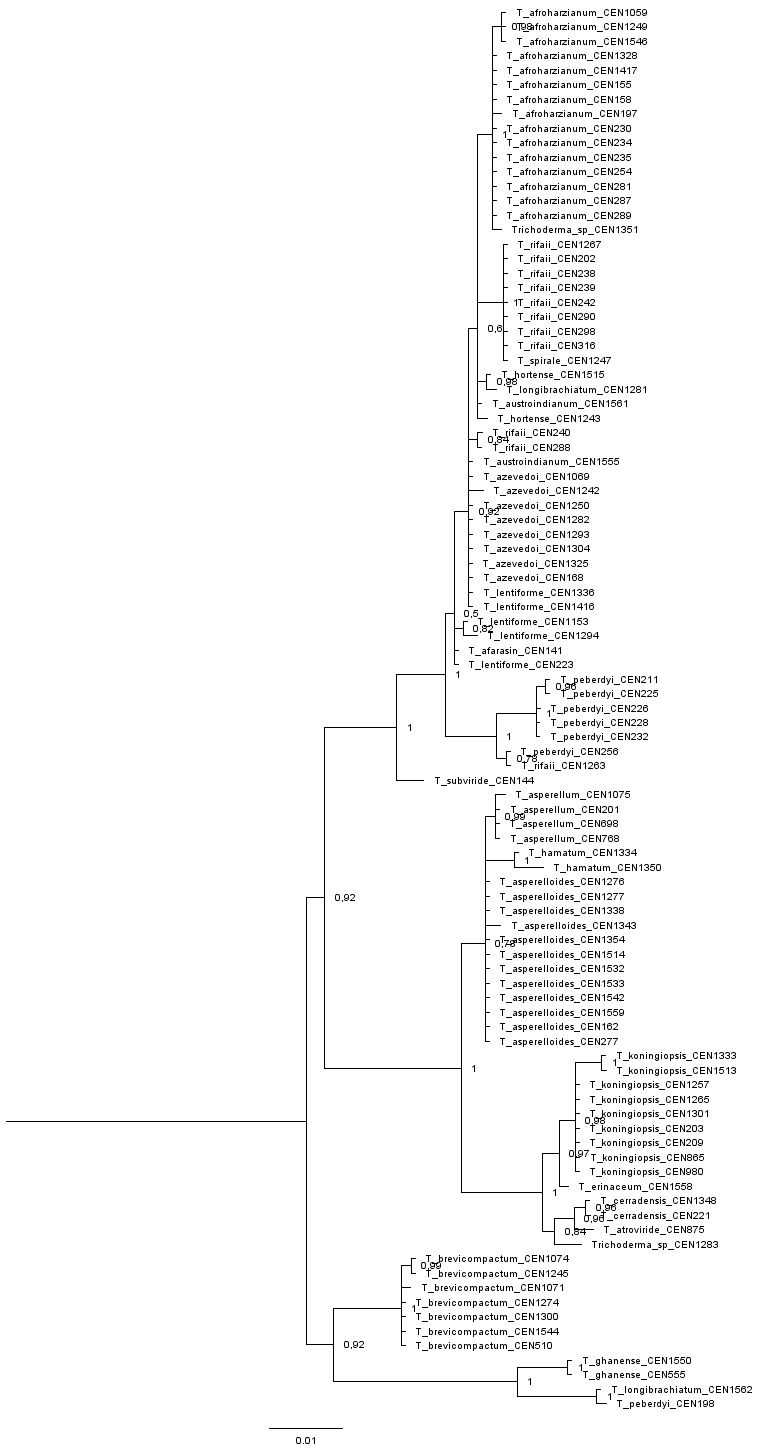
**

**Supplementary Material 27.** Bayesian phylogenetic tree based on RPB2 sequences of all strains of this study. Bayesian posterior probability values are indicated at the nodes, and the scale bar represents the number of expected changes per site.

**
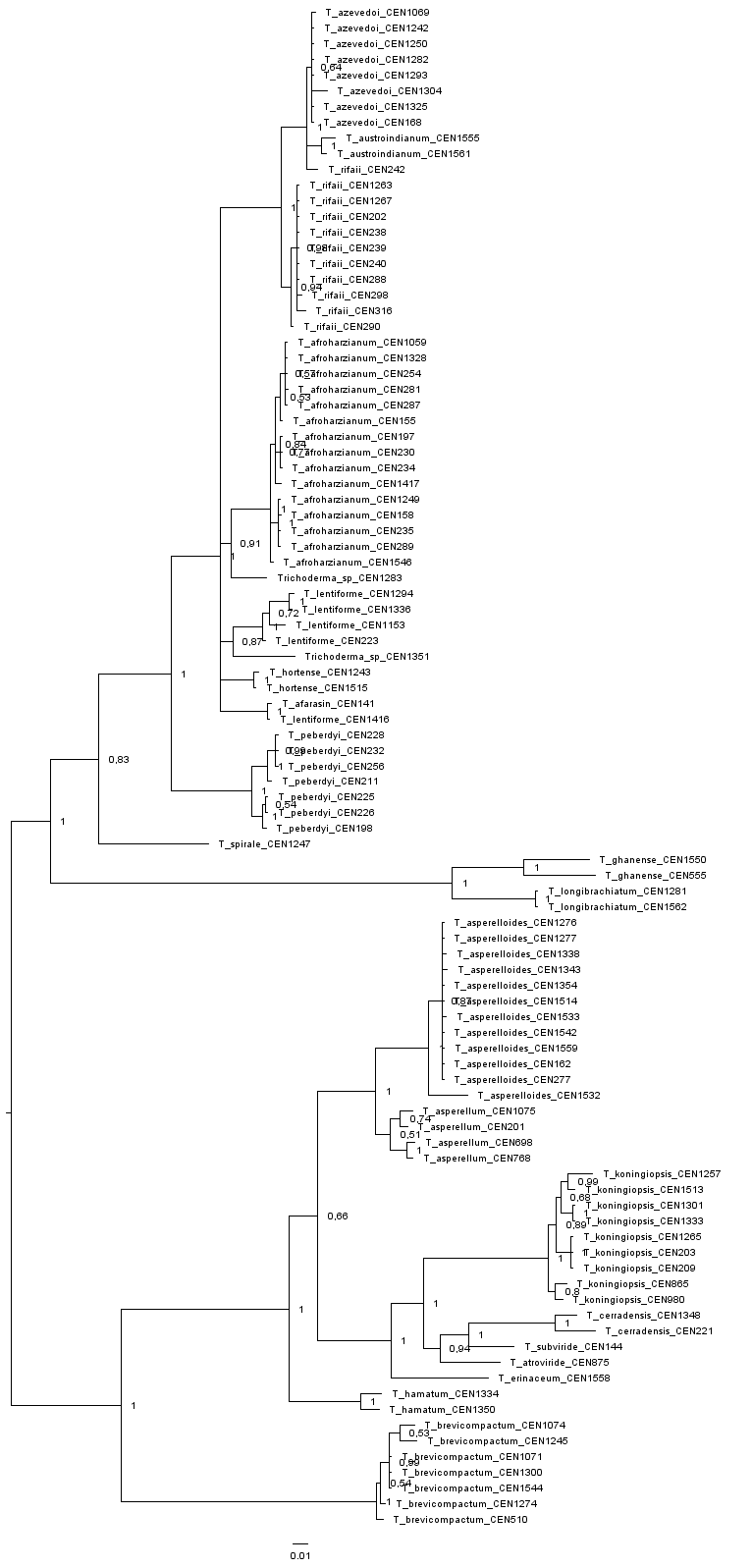
**

**Supplementary Material 28.** Bayesian phylogenetic tree based on ITS sequences of all strains of this study. Bayesian posterior probability values are indicated at the nodes, and the scale bar represents the number of expected changes per site.

**
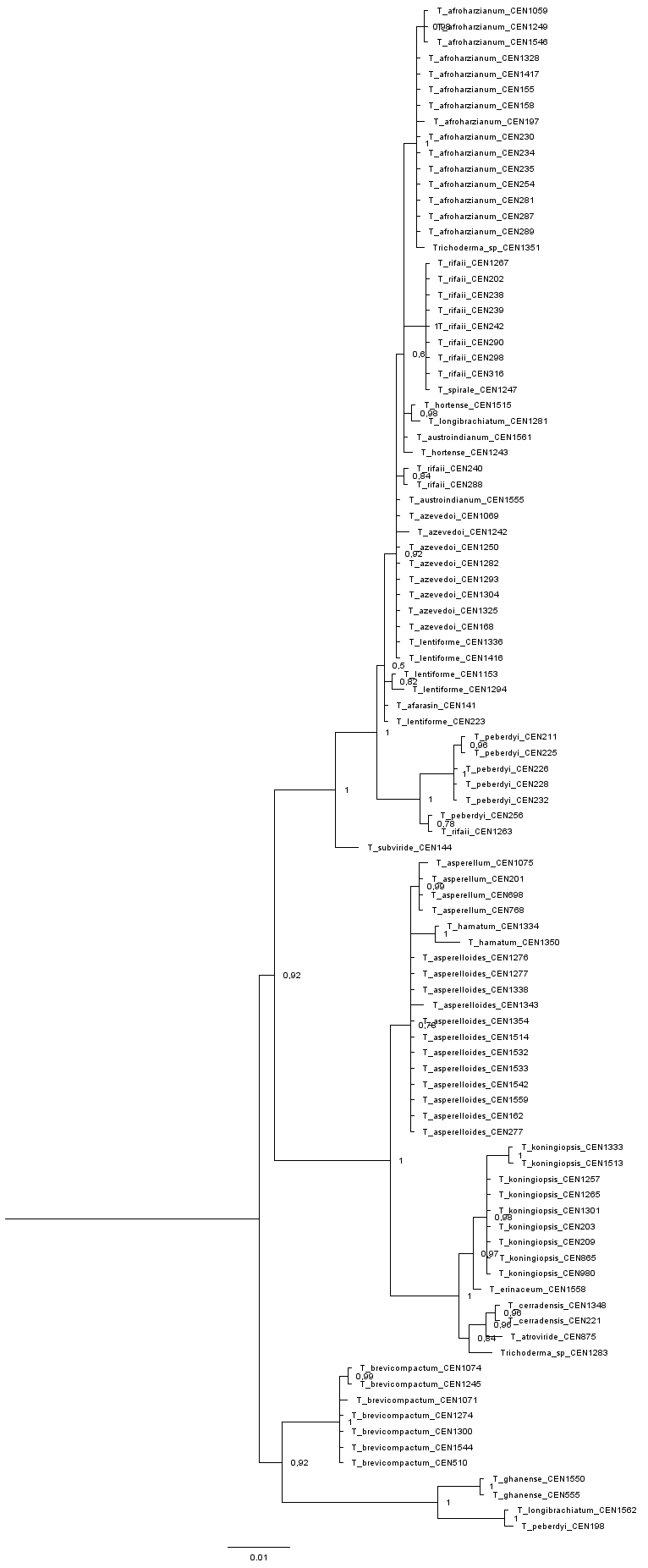
**

**Supplementary Material 29.** Bayesian phylogenetic tree based on CAL sequences of all strains of this study. Bayesian posterior probability values are indicated at the nodes, and the scale bar represents the number of expected changes per site.

**
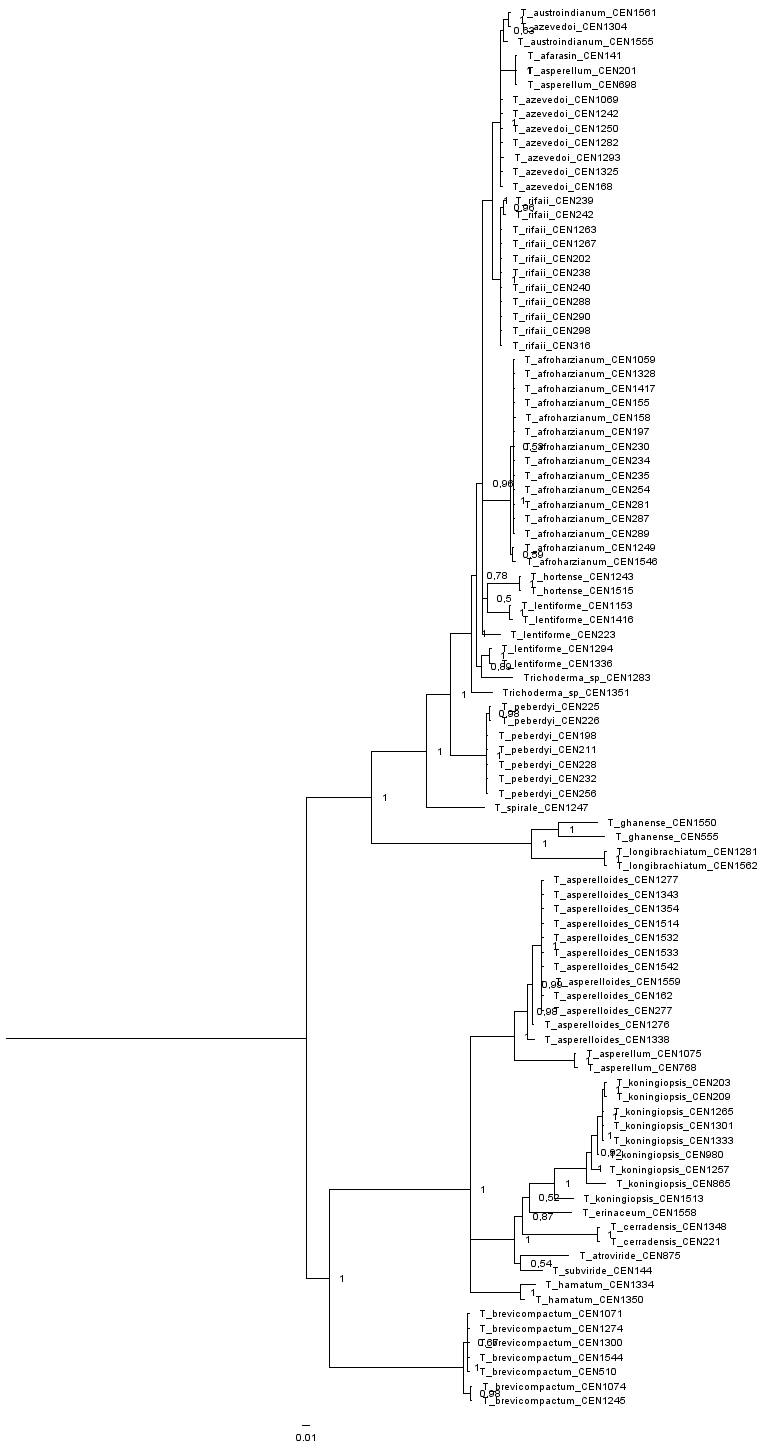
**

**Supplementary Material 30.** Bayesian phylogenetic tree based on ACT sequences of all strains of this study. Bayesian posterior probability values are indicated at the nodes, and the scale bar represents the number of expected changes per site.


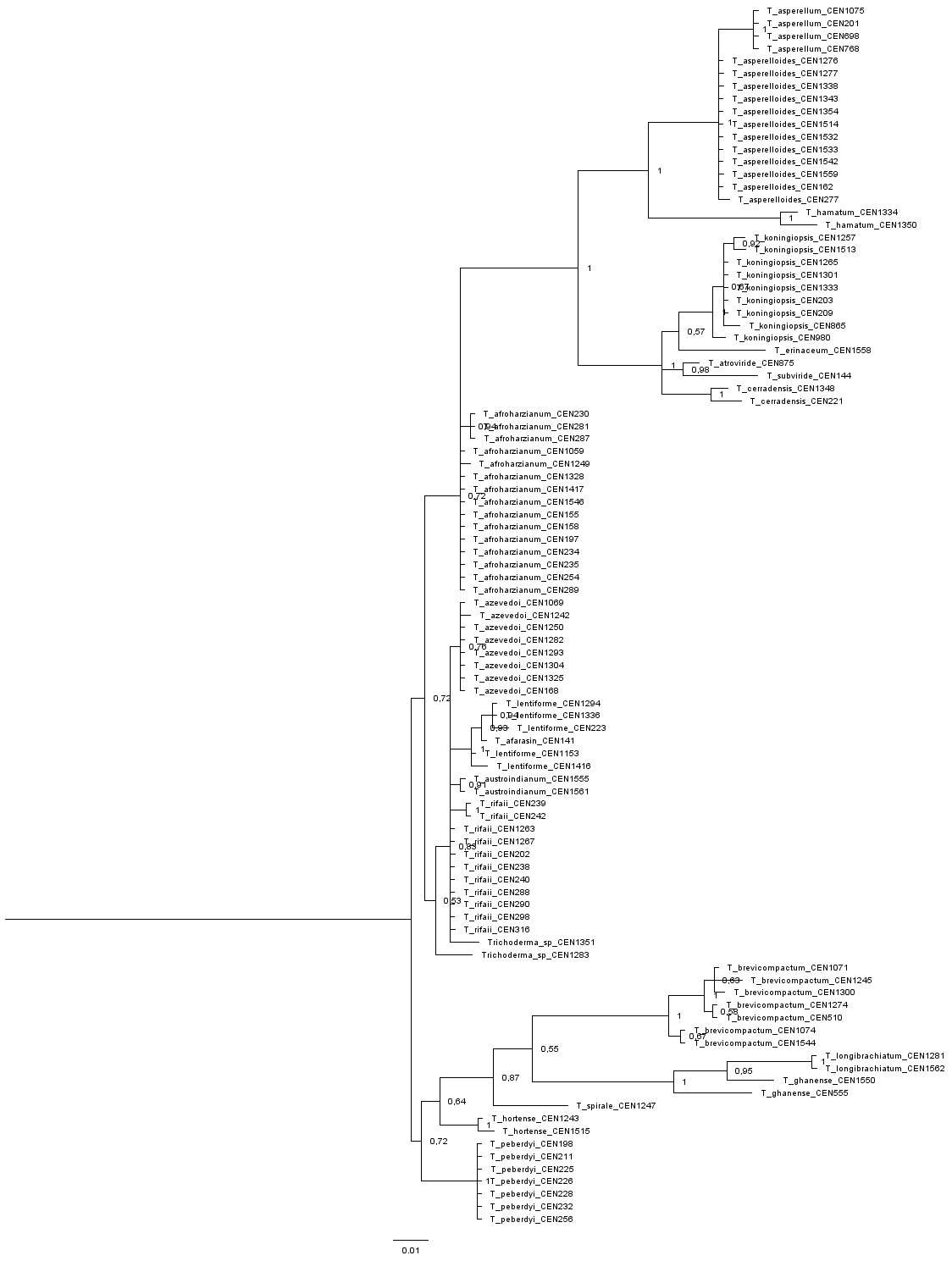

Supplement: Supplementary file 1 [file Data_Sheet_1.docx]
